# Supplementary material for: Water-dispersible X-ray scintillators enabling coating and blending with polymer materials for multiple applications
Source: Nat Commun. 2024 Mar 6;15:2055. doi: 10.1038/s41467-024-46287-8 (PMC10917805; doi:10.1038/s41467-024-46287-8)
Supplement: Supplementary file 1 — Supplementary Information [file 41467_2024_46287_MOESM1_ESM.pdf]

Supplementary Information

Water-dispersible X-ray scintillators enabling coating and blending with polymer materials for multiple applications

Hailei Zhang[1,2,*], Bo Zhang[1], Chongyang Cai[3], Kaiming Zhang[2], Yu Wang[1], Yuan Wang[1], Yanmin Yang[3,*], Yonggang Wu[1], Xinwu Ba[1], and Richard Hoogenboom[2,*]

[1]College of Chemistry & Materials Science, Hebei University, 180 Wusi Road, 071002 Baoding, China; [2]Supramolecular Chemistry Group, Centre of Macromolecular Chemistry (CMaC), Department of Organic and Macromolecular Chemistry, Ghent University, Krijgslaan 281-S4, 9000 Gent, Belgium; [3]College of Physics Science and Technology, Hebei University, 180 Wusi Road, 071002 Baoding, China;

E-mails:
zhanghailei@hbu.edu.cn; mihuyym@163.com;
richard.hoogenboom@ugent.be

Contents

Supplementary Note 1: Materials 4
Supplementary Note 2: Characterization 4
Supplementary Method 1: Synthesis of Tb^{3+}-doped $Na_5Lu_9F_{32}$ 6
Supplementary Method 2: Synthesis of Tb^{3+}-doped $NaLuF_4$ 6
Supplementary Method 3: Light yield calculation 6
Supplementary Method 4: Cell culture and viability test 7
Supplementary Discussion 1: Synthesis and characterization 8
Supplementary Discussion 2: Exploration of the difference in emission behaviors 9
Supplementary Discussion 3: Cell viability test 11
Supplementary Discussion 4: Comparisons with reported X-ray scintillators 11
Supplementary Discussion 5: Investigation of the penetration abilities 13
Supplementary Discussion 6: Different excitation–emission mechanisms 13
Supplementary Figure 1. Characterizations of Tb^{3+}-doped $Na_5Lu_9F_{32}$. 15
Supplementary Figure 2. Characterizations of Tb^{3+}-doped $NaLuF_4$. 16
Supplementary Figure 3. Photographs of obtained scintillators 17
Supplementary Figure 4. Nuclear magnetic resonance results. 17
Supplementary Figure 5. Fourier transform infrared spectroscopy results. 18
Supplementary Figure 6. Thermal gravimetric results. 19
Supplementary Figure 7. X-ray powder diffraction results. 20
Supplementary Figure 8. Micromorphology characterizations of pristine halloysite nanotubes. 21
Supplementary Figure 9. High-angle annular dark-field scanning transmission electron microscopy image. 21
Supplementary Figure 10. Radioluminescence spectra for calculating light yields.. 22
Supplementary Figure 11. Gravimetrical method results.. 23
Supplementary Figure 12. Elemental analysis on the cross-section.. 23
Supplementary Figure 13. Radioluminescence spectra in stability test. 24
Supplementary Figure 14. Photographs of the obtained composite foams. 24
Supplementary Figure 15. Flexible X-ray scintillator screen.. 25
Supplementary Figure 16. Characterization of oleic acid-modified Tb^{3+}-doped $Na_5Lu_9F_{32}$. 26
Supplementary Figure 17. Rheological sweep curves of the multi-layer hydrogel. 27
Supplementary Figure 18. Size distribution in aqueous solution.. 27
Supplementary Figure 19. Afterglow results. 28
Supplementary Figure 20. Thermoluminescence spectra 28
Supplementary Figure 21. Cell viability data. 29
Supplementary Table 1. Comparisons between the obtained $Na_5Lu_9F_{32}$:Tb^{3+}-anchored halloysite nanotubes (HNTs@$Na_5Lu_9F_{32}$:Tb^{3+}) with non-perovskite-type X-ray scintillators that have been reported in literature. 30
Supplementary Table 2. Comparisons between the obtained $Na_5Lu_9F_{32}$:Tb^{3+}-anchored halloysite nanotubes (HNTs@$Na_5Lu_9F_{32}$:Tb^{3+}) with perovskite-based X-ray scintillators that have been reported in literature. 31

Supplementary Table 3. Summary and analysis of the atom percentage of Al, Si, and F in the cross-section of $Na_5Lu_9F_{32}:Tb^{3+}$-anchored halloysite nanotubes coated polyurethane foam (HNTs@$Na_5Lu_9F_{32}:Tb^{3+}$@PUF) (8.0 cm × 8.0 cm ×8.0 cm) from SEM-EDS 33
Supplementary Table 4. Summary of the materials exhibit different emission behaviors between UV/Vis-induced photoluminescence and X-ray-induced radioluminescence. 34
Supplementary References 35

Supplementary Note 1: Materials

Halloysite nanotubes (HNTs) were obtained from GuangZhou Shinshi Metallurgy and Chemical Co., Ltd and purified in alkali water solution (pH = ca. 9, adjusted by sodium hydroxide). Sodium hexametaphosphate (10%, w/v) was added to the solution and magnetically stirred for 6 h under room temperature. The impurities can be removed under by centrifugation under 5,000 rpm. Then the supernatant is further centrifugated under 7,000 rpm to afford the purified HNTs. 1,4-Phenylenebisdiboronic acid, 4-bromobenzophenone, and 4,4'-dibromobenzil were obtained from Sigma-Aldrich. Bis(pinacolato)diboron was obtained from Soochiral Chemical Science & Technology Co., Ltd. Pd(dppf)Cl_2 was obtained from Shanghai Opdisi Chemical Technology Co., Ltd. 3-Aminopropyltriethoxysilane (KH550®), carboxymethylcellulose sodium (CMC-Na), and $Lu(NO_3)_3·6H_2O$ were purchased from Shanghai Aladdin Biochemical Technology Co., Ltd. Citric acid (CA), oleic acid (OA), and NH_4F were obtained from Shanghai Macklin Biochemical Co., Ltd. $Tb(NO_3)·6H_2O$ were obtained from Heowns Biochem Technologies. Polyurethane foam (PUF) was purchased from Minnesota Mining and Manufacturing. Epoxy resin E51 and polyamide curing agent (JDF-650) were purchased from the Alibaba Co., Ltd. Polyvinyl alcohol (PVA, M_n = 1,750 ± 50 g/mol) was obtained from Tianjing Damao Chemical Reagent Factory. Distilled water was used throughout the study. Aminated HNTs (HNTs-NH_2) is prepared by treating HNTs with KH550®, details can be found in our previous study.[1] The synthesis of tetraphenylethylene (TPE)-containing crosslinker (**M3**) has been reported in our previous study.[2]

Supplementary Note 2: Characterization

The X-ray-excited radioluminescence (RL) spectra were recorded using an Andor SR-500i spectrometer (Andor Technology Co. Belfast, UK) equipped with a Hamamatsu R928 photomultiplier.

The thermoluminescence spectra were acquired on a self-assembled system comprising high-precision thermal stages (THMS600, British Linkam Scientific Instruments) and an Andor SR-500i spectrometer (Andor Technology Co. Belfast, UK) at a fixed heating rate of 3 °C s^{-1} between room temperature and 350 °C.

Thermal gravimetric analysis (TGA) was performed on a TA Q50 thermal gravimetric analyzer (TA Instruments, US) under a nitrogen flow. Accurately weighted amounts of samples were heated at a scanning rate of 10 °C min^{-1} from room temperature to 800 °C.

X-ray photoelectron spectroscopy (XPS) was carried out on a Thermo Scientific ESCALab 250Xi (Thermo Fisher Scientific, US) using 200 W monochromated Al K Alpha radiation. The 500 μm X-ray spot was used for XPS analysis. The base pressure in the analysis chamber was about 3 × 10^{-10} mbar. Typically, the hydrocarbon C 1s line at 284.8 eV from adventitious carbon was used for energy referencing. Other acquisition parameters: Number of Scans: 5; Lens Mode: Standard; Analyser Mode: CAE: Pass Energy 30.0 eV; Energy

Step Size: 0.050 eV; Number of Energy Steps: ca. 400.

X-ray power diffraction (XRD) patterns were recorded on a D8 ADVANCE X-ray powder diffractometer system (Bruker Corporation, German) using a tube voltage of 40 kV, a current of 40 mA, a range of 10° to 90° and a step size of 0.06°.

Fourier transform infrared spectroscopy (FTIR) spectra were recorded in the region of 4,000-400 cm^{-1} for each sample on a Nicolet iS10 Fourier transform infrared spectrophotometer (Thermo Fisher Scientific, US). Samples were previously grounded and mixed thoroughly with KBr. The spectrum for each sample was obtained from averaging 32 scans over the selected wavenumber range.

The morphological characterizations, including transmission electron microscope (TEM) observations, energy dispersive X-ray spectroscopy (EDX) maps and high-angle annular dark-field (HAADF) Scanning TEM (STEM) images were performed by using a F200x FEI TalosF200x scanning transmission electron microscope (Thermo Fisher Scientific, US) or a Phenom ProX Desktop scanning transmission electron microscope (Phenom, Netherlands).

Scanning electron microscope (SEM) observation and elemental distributions were conducted on a TESCAN MIRA LMS field emission scanning electron microscopy (TESCAN, Czech).

^{13}C solid-state nuclear magnetic resonance (NMR) spectra were obtained on a Bruker 400 M spectrometer (Bruker Corporation, German). Solution ^{13}C NMR and ^{1}H NMR spectra were performed on a Bruker AV600 spectrometer (Bruker Corporation, German) or a QOne WNMR-I-400MHz spectrometer (Zhongke-Niujin, China) using tetramethylsilane as internal standard.

AR2,000ex-type rotational rheometer (TA, US) was used to investigate the rheological properties.

The dynamic light scattering (DLS) measurements were performed by using a commercialized spectrometer from Brookhaven BI-200SM Goniometer equipping a 17 mW He−Ne laser (633 nm). A Laplace inversion program was used to process the data to obtain the effective diameter and polydispersity index (PDI).

Stress-strain measurements were performed on a universal testing machine (WDW-02). The tensile measurements were performed using a pulling speed of 10 mm min^{-1} until sample failure. The stress σ was recorded as a function of strain ε.

A WD-2A stability test instrument was used to evaluate the stability of HNTs@$Na_5Lu_9F_{32}$:Tb^{3+}@PUF and of the X-ray scintillator screen (Temperature: 60 ± 0.5°C; Light intensity: 2,000 lx, white; Humidity: 50 ± 4%). The samples were placed in stability test instrument for 30 days. The radioluminescence intensity of the samples before and after the test was recorded to evaluate the stability.

Supplementary Method 1: Synthesis of Tb^{3+}-doped $Na_5Lu_9F_{32}$

$NaNO_3$ (0.51 g, 6.0 mmol), $Lu(NO_3)_3{\cdot}6H_2O$ (4.69 g, 10.0 mmol), and $Tb(NO_3)_3{\cdot}6H_2O$ (0.45 g, 1.0 mmol) were dissolved into 10 mL water and then added into 10 mL water solution containing citric acid (0.30 g, 1.56 mmol). NaF (1.48 g, 35 mmol) was added into the system and then the pH value was adjusted to 5.0 by addition of nitric acid. The system was stirred at a low speed of 200 rpm for 30 min to achieve a homogeneous suspension. The agitation was continued for another 30 min. The obtained suspension was introduced into a hydrothermal reactor with a filling rate of 70% and then heated at 180°C for 24 h. After cooling to room temperature, the solution was added into 200 mL absolute ethanol. The residue was collected by centrifugation and then washed with water and ethanol. The obtained solid was dried in vacuum to afford Tb^{3+}-doped $Na_5Lu_9F_{32}$ ($Na_5Lu_9F_{32}$:Tb^{3+}) as white solid (1.26 g).

Oleic acid-modified $Na_5Lu_9F_{32}$:Tb^{3+} (OA@$Na_5Lu_9F_{32}$:Tb^{3+}) was prepared following a typical method in the literature for the surface modification of inorganic nanoparticles.[3] Briefly, 500 mg of $Na_5Lu_9F_{32}$:Tb^{3+} was added into 7.5 mL oleic acid and heated at 80 °C for 1 h. After cooling to room temperature, the residue was collected by centrifugation and then washed with ethanol and acetone. The obtained solid was dried in vacuum to afford OA@$Na_5Lu_9F_{32}$:Tb^{3+} as gray solid.

Supplementary Method 2: Synthesis of Tb^{3+}-doped $NaLuF_4$

NaOH (0.625 g, 15.6 mmol) was dissolved in 12 mL ethanol. Oleic acid (18 mL, 56.7 mmol) was dropwise added into the NaOH solution and then stirred for 0.5 h. NH_4F (0.37 g, 10 mmol), $Tb(NO_3)_3{\cdot}6H_2O$ (45 mg, 0.10 mmol), and $Lu(NO_3)_3{\cdot}6H_2O$ (0.422g, 0.90 mmol) were added into the mixture and stirred for another 0.5 h, followed by transferred into to hydrothermal synthesis reactor equipped with polytetrafluoroethylene liner. The solution was then heated at 190°C for 20 h. The residue was collected by certification and then washed by water, ethanol, and acetone. After dried in a vacuum, the product Tb^{3+}-doped $NaLuF_4$ ($NaLuF_4$:Tb^{3+}) can be obtained as white solid. $NaLuF_4$:Tb^{3+} with different doping concentrations of Tb^{3+} (0.1%, 1%, 5%, 10%, and 15%) was prepared by changing the addition amount of $Lu(NO_3)_3{\cdot}6H_2O$ and $Tb(NO_3)_3{\cdot}6H_2O$.

Supplementary Method 3: Light yield calculation

The light yield of the obtained scintillators was measured according to the reference.[4] LuAG:Ce scintillator of light yield (*LY*) 25,000 photons MeV^{-1} was used as a reference to calibrate the light yields of our scintillators. Our scintillators and the reference wafer were set at the same position to measure the RL spectra. The *LY* can be calculated from the following Supplementary Eq.1:

$$LY_{\mathrm{s}} = LY_{\mathrm{LuAG:Ce}} \times \frac{S_{\mathrm{s}} \times a_{\mathrm{LuAG:Ce}}}{S_{\mathrm{LuAG:Ce}} \times a_{\mathrm{s}}} \quad \text{(Supplementary Eq.1)}$$

where $a_{LuAG:Ce}$ and a_s are the X-ray attenuation coefficiency, $S_{LuAG:Ce}$ and S_s are the integral intensities, and $LY_{LuAG:Ce}$ is the light yield of LuAG:Ce, 25,000 photons MeV^{-1}.

The X-ray attenuation coefficient was calculated by measuring I_0 and I (X-ray intensities of the incident and transmitted beams). It is possible to determine the X-ray attenuation coefficient a as:[5]

$$a = -\frac{1}{x}\ln\frac{I}{I_0} \qquad \text{(Supplementary Eq. 2)}$$

where x is the thickness of the sample. I_0 and I represent the X-ray intensities of the incident and transmitted beams, respectively.

The volume and thickness for all samples, cylindrical in shape, were 0.0157 cm^3 and 0.2 mm, respectively. The density of standard sample (LuAG:Ce), $Na_5Lu_9F_{32}:Tb^{3+}$, and HNTs@$Na_5Lu_9F_{32}:Tb^{3+}$ were calculated as 6.72, 3.76, and 3.63 g cm^{-3}, respectively. A 30 mA 40 kV X-ray irradiator is used in measuring the X-ray attenuation coefficient and light yields.

Supplementary Method 4: Cell culture and viability test

Mouse fibroblast L929 cells were purchased from the Cell Bank of the *Shanghai* Chinese Academy of Science. The cells were maintained in Dulbecco's modified Eagle's medium (DMEM) and supplemented with 10% fetal bovine serum (FBS) within a humidified environment (37°C) containing 5% CO_2 and 1% penicillin/streptomycin antibiotics. In the cell viability test, mouse fibroblast L929 cells were seeded in 96-wellplate and then incubated with HNTs@ $Na_5Lu_9F_{32}:Tb^{3+}$ at 37 °C with the concertation ranging from 1 to 1,000 μg mL^{-1}. After 24 and 48 h, methyl thiazolyl tetrazolium (MTT) solution was added to each well, another incubation process was continued for 4 h. Then the medium was removed and 100 μL DMSO was added. After homogenizing well, the absorbance of each well was measured at 570 nm by the ELX-800 microplate reader (ELISA Reader). Wells without the addition of samples were used as blank control. The cell viability (%) was calculated by the absorbance percentage of test to control.

Supplementary Discussion 1: Synthesis and characterization

The weak signal at 179.7 ppm in the solid-state ^{13}C NMR spectrum of the HNTs-CA, depicted in Supplementary Fig. 4, corresponds to the carbon atoms in the C=O groups of CA, as evidenced by the ^{13}C NMR spectrum of citric acid in D_2O (176.7 and 173.4 ppm). The peaks at 75.6 and 41.7 ppm can be attributed to the quaternary carbon atom and the carbon atoms in the $–CH_2$- groups of citric acid. The pristine HNTs do not exhibit these signals in the ^{13}C NMR spectrum.[6] The resonances at 21.0 and 9.0 ppm correspond to the carbon atoms of the 3-aminopropyltriethoxysilane unit. FTIR analysis (detailed shown in Supplementary Fig. 5) was performed to reveal the presence of the citric acid in the HNTs-CA by comparing it with the spectra of pristine HNTs, HNTs-NH_2, and CA. The broad band from 4,000 to 3,000 cm^{-1} illustrates the characteristic stretching from the HNTs resulting from the -OH groups in the aluminosilicate skeletons. In addition, the peaks at 3,696 and 3,618 cm^{-1} can be attributed to the -OH groups in different chemical environments of the inner surface and the curled layer, which is a typical characteristic in HNTs. The stretching vibration around 1,030 cm^{-1} is the characteristic band of in-plane Si–O–Si bonds. These characteristic peaks are preserved in the FTIR spectra of the HNTs-CA and HNTs-NH_2, implying that the basic composition of the HNTs remained intact during the modification processes. Moreover, a new peak emerged at 1,727 cm^{-1}, suggesting the presence of the -C=O groups of the citric acid moieties, thereby confirming the successful introduction of the citric acid onto the HNTs. The TGA results depicted in Supplementary Fig. 6 indicate that the grafting degrees of HNTs-NH_2 and HNTs-CA correspond to ca. 2.6 wt% and 4.4 wt%, respectively, indicating that ~50% of the -NH_2 groups were modified.

$Na_5Lu_9F_{32}:Tb^{3+}$ and $NaLuF_4:Tb^{3+}$ were synthesized via a solvothermal method by using different chelating agent. The peaks in X-ray diffraction (XRD) patterns of $Na_5Lu_9F_{32}:Tb^{3+}$ and $NaLuF_4:Tb^{3+}$ shown in Supplementary Figs. 1a and 2a match well with the standards. The XRD results indicate that the obtained $Na_5Lu_9F_{32}:Tb^{3+}$ obeys to the character of cubic lattice system (a = b = c = 5.464 Å) and the density is calculated as 6.14 g cm^{-3}. Size distributions of HNTs, HNTs@$Na_5Lu_9F_{32}:Tb^{3+}$, and $Na_5Lu_9F_{32}:Tb^{3+}$ can be found in Supplementary Fig. 18.

SEM and TEM were used to reveal the micromorphology characters (Supplementary Figs. 1c-f, 1i, 2c, 2d, 2f, and 2g). The results show that the obtained $Na_5Lu_9F_{32}:Tb^{3+}$ exhibits unregular sphere-like particles. For $NaLuF_4:Tb^{3+}$, regular hexagonal phase can be obtained. XPS was used to reveal the chemical composition of $Na_5Lu_9F_{32}:Tb^{3+}$ and $NaLuF_4:Tb^{3+}$. The presence of sodium, lutetium, fluorine, and terbium is demonstrated based on the peaks around 1,277 eV (Tb 3d3), 1,243 eV (Tb 3d4), 1,072 eV (Na 1s), 686 eV (F 1s), and 198 eV (Lu 4d5), respectively. Both of the RL spectra of $Na_5Lu_9F_{32}:Tb^{3+}$ and $NaLuF_4:Tb^{3+}$ features four emission peaks at 489, 544, 585, and 620 nm. The X-ray-induced long persistent luminescence properties were investigated. The persistent luminescence decay curves monitored at 544 nm

after irradiation by an X-ray irradiator for 10 min were recorded, as shown in Supplementary Fig. 2h. The afterglow intensity decreased quickly in the first hour and then decayed slowly. Even after 4×10^3 s, the afterglow can even be detected. $Na_5Lu_9F_{32}:Tb^{3+}$ cannot any persistent luminescence behaviors. The decay time of $Na_5Lu_9F_{32}:Tb^{3+}$ is found less than 1 s, which exhibits a synchronous RL behavior following the “On-Off” switching of X-ray irradiation (Supplementary Fig. 1h).

OA@$Na_5Lu_9F_{32}:Tb^{3+}$ was also characterized by XPS (Supplementary Fig. 16a-16d). The peaks assigned to Tb 3d3, Tb 3d4, Na 1s, F 1s, and Lu 4d5 can also detected. The intensity of C 1s is much higher than that of $Na_5Lu_9F_{32}:Tb^{3+}$, suggesting the oleic acid moieties have been anchored on the surface of the nanoparticles. The obtained OA@$Na_5Lu_9F_{32}:Tb^{3+}$ shows a better water-dispersibility (Supplementary Fig. 15e) than pristine $Na_5Lu_9F_{32}:Tb^{3+}$.

Supplementary Discussion 2: Exploration of the difference in emission behaviors

The mechanism of the X-ray-induced persistent luminescence phenomenon, also known as afterglow, is still under investigation and debate. Up to now, several underlying mechanisms have been put forward to explain X-ray-induced persistent luminescence, including the hole trapping-detrapping model, the electron trapping-detrapping model, and the quantum tunneling model.[7-9] Although these models could explain some observed phenomena, there are flaws in these models and some key points still remain unclear.

To our best knowledge, persistent luminescence is governed by the following aspects: whether the excitation can effectively charge energy, whether the heat can effectively release charge carriers, and whether the luminescent center can effectively bind charge carriers and produce emissions.[10,11] Previous research has mainly concentrated on the former two aspects, while rarely researchers focused on the third topic. Conventionally, the binding ability between lanthanide ions and charge carriers is viewed to be affected by the inherent arrangement of the electrons and ionization energies of lanthanides.[12,13] Ce^{3+}, Pr^{3+}, and Dy^{3+} can easily bind traps, while Eu^{3+}, Yb^{3+}, Sm^{3+}, and Tm^{3+} are more likely to bind electrons. The persistent luminescent abilities from the above lanthanides can be explained by this hypothesis. However, the persistent luminescence from Gd^{3+} can not be explained by this model because Gd^{3+} can not easily bind either electrons or traps. The Gd^{3+} doped $ScPO_4$ shows obvious X-ray-induced persistent luminescence, while the persistent luminescence ability seriously declines for Gd^{3+} doped YPO_4 and $LuPO_4$. The persistent luminescence is difficult to be detected in Gd^{3+} doped $LaPO_4$ with the same doping concentration.[14] The different persistent luminescence behavior in different hosts raised our concern and inspired us to explore alternative models.

To break the limitation, we proposed a mechanism for the trivalent lanthanides' persistent luminescence based on abundant experiments and

analysis in a previous study.[14] According to the mechanism, the trivalent lanthanides as isoelectronic traps are expected to eventually bind excitons, and this binding ability is not only related to the inherent arrangement of the electrons of the trivalent lanthanides, but also to the extrinsic anion coordination and cation substitution in the host lattices. Following this way, the persistent luminescent that came from Gd^{3+} can be well explained and the persistent luminescent ability can be regulated by changing the coordinated anions and substituted cations in the host lattices. The excitons in such materials transfer their recombination energy to the trivalent lanthanides, followed by the generation of persistent luminescence from the trivalent lanthanides.

In this study, X-ray is used as the excitation, which can charge energy to nearly all kinds of hosts. The factors that decide the persistent luminescence properties and eventually lead to the presence or absence of afterglows may be attributed to the following aspects: character of the luminescent center, character of hosts (coordinated anions, substituted cations, type of coordinated linkage, symmetry, etc.), and doping concentration of lanthanides.

$Na_5Lu_9F_{32}$:Tb^{3+} and $NaLuF_4$:Tb^{3+} have the same luminescent center and therefore display identically the same emission peaks in X-ray-excited RL spectra. To further evaluate the influence of the doping concentration of Tb^{3+} on the persistent luminescence properties, we prepared $NaLuF_4$:Tb^{3+} with different doping concentrations of Tb^{3+} (0.1%, 1%, 5%, 10%, and 15%). The afterglow intensity from $NaLuF_4$:Tb^{3+} monitored at 544 nm was recorded in Supplementary Fig. 19. All of the samples display typical afterglow curves and the optimal afterglow property can be found in the case of 10%. The results suggest that the persistent luminescence properties can be affected by the doping concentration of Tb^{3+}, while it is not the crucial factor to decide the presence or absence of afterglow. Therefore, the difference between the persistent RL from $NaLuF_4$:Tb^{3+} and synchronous RL behavior from $Na_5Lu_9F_{32}$:Tb^{3+} following the “On-Off” switching of X-ray irradiation should be attributed to the difference in the structure of the hosts.

$Na_5Lu_9F_{32}$ and $NaLuF_4$ share the same elements. The major difference between them is the lattice types. According to the XRD results, $Na_5Lu_9F_{32}$ obeys the character of a cubic lattice system, while the lattice type of $NaLuF_4$ should be classified into a hexagonal phase. It has been reported that the difference between cubic and hexagonal lattice within the crystals bearing the same elements usually possess different binding energies,[15,16] which eventually gives rise to different optical properties.[17,18] Liu’s group has demonstrated that the hexagonal-phased lattices composed of Na, Ln (lanthanide), and F are more suitable for achieving energy transfer and energy migration, as opposed to the cubic-phased counterpart.[19] On the other hand, the cubic lattice composed of Na, Ln, and F has eight coordinated holes, which is easily contribute to nonradiative quenching through reduced migration of the exciton energy.[18] Therefore, the hexagonal-phased lattices in $NaLuF_4$ may hold more efficient energy transfer and thereby result in sufficient recombination

energy that can be transferred to the doped Tb^{3+}, followed by the generation of persistent luminescence from the trivalent lanthanide.

The difference in persistent luminescence properties between cubic and hexagonal lattices has also received attention by other researchers. Tang *et al.* reported a similar phenomenon that only the excitation through the hexagonal-phased $CsCdCl_3$ host could give persistent emission, while the persistent emission cannot be observed for the cubic-phased $CsCdCl_3$ host.[20] They also attributed the difference in persistent luminescence properties to the efficient energy transfer in hexagonal lattices, matching well with our findings.

Otherwise, the difference in lattice types may result in different trap properties. Generally, the persistent performance of materials is closely related to the thermally-stimulated gradual release of charge carriers which are immobilized in the trap centers.[21] The persistent properties are highly determined by the trap properties, which can be investigated by the analysis of the thermoluminescence (TL) spectra.[22] The TL intensity reflects the charge carrier concentration captured at the trap.[21,23] We also investigate the TL behaviors of $Na_5Lu_9F_{32}:Tb^{3+}$ and $NaLuF_4:Tb^{3+}$ after the X-ray irradiation. As shown in Supplementary Fig. 20, the TL spectrum of $NaLuF_4:Tb^{3+}$ displays a strong peak centered at 187 °C, while $Na_5Lu_9F_{32}:Tb^{3+}$ can not show any TL behavior. The results suggest that the traps in $NaLuF_4:Tb^{3+}$ exhibit much higher binding ability to charge carrier than that of $Na_5Lu_9F_{32}:Tb^{3+}$, which may also contribute to the difference between the persistent RL from $NaLuF_4:Tb^{3+}$ and synchronous RL behavior from $Na_5Lu_9F_{32}:Tb^{3+}$.

Supplementary Discussion 3: Cell viability test

The cell viability of mouse fibroblast L929 cells remained above 90% even at relatively high concentrations (1,000 μg mL^{-1}) after 24 h and 48 h of treatment by HNTs@$Na_5Lu_9F_{32}:Tb^{3+}$ (Supplementary Fig. 21) The results indicate that the obtained HNTs@$Na_5Lu_9F_{32}:Tb^{3+}$ exhibits negligible biological toxicity on mouse fibroblast L929 cells.

Supplementary Discussion 4: Comparisons with reported X-ray scintillators

The comparisons between HNTs@$Na_5Lu_9F_{32}:Tb^{3+}$ with X-ray scintillators that have been reported in the literature were made in Supplementary Tables 1 and 2. The items of processing method, materials types, application fields, light yield, and emission wavelength (λ_{em}) were summarized.

Supplementary Table 1 shows the comparisons between the obtained HNTs@$Na_5Lu_9F_{32}:Tb^{3+}$ with non-perovskite-type X-ray scintillators that have been reported in literature. It should be noted that the processing of X-ray scintillators into macroscopic materials, especially polymer composites, is still difficult to get rid of the use of organic solvent, high temperatures, or harsh conditions for crystal growth in literature. The obtained HNTs@$Na_5Lu_9F_{32}:Tb^{3+}$ in our study is easily processable as aqueous dispersion to develop composite

foams, flexible/hard screens, and hydrogels. The light yield of the obtained HNTs@$Na_5Lu_9F_{32}$:Tb^{3+} was estimated to be 12,300 photons MeV^{-1}, which is higher than well-known commercial scintillators including BaF_2 (1,400 photons MeV^{-1}), $Bi_4Ge_3O_{12}$ (BGO, 8,500 photons MeV^{-1}), and Gd_2SiO_5 (GSO):Ce (7,000 photons MeV^{-1}). Though some single crystals, such as LYSO:Ce and $LaBr_3$:Ce, exhibit higher light yields, the high fabrication cost, harsh growth conditions, and non-flexibility limit their application to conventional hard devices.

Supplementary Table 2 shows the comparisons between the obtained HNTs@$Na_5Lu_9F_{32}$:Tb^{3+} with recently reported perovskite-type X-ray scintillators. Similar to the former non-perovskite-type cases, perovskite-type X-ray scintillators also suffer from the above-mentioned limitations. Concerns are also raised on the use of perovskite in view of the presence of heavy metals, especially lead. Moreover, the X-ray RL behavior of perovskite-based X-ray scintillators is usually affected by the strong thermal quenching effects even under room temperature because of the low band gaps. Take $CsPbBr_3$ for an example, it holds a high light yield of ~50,000 photons MeV^{-1} at 7K while the light yield is determined as <500 photons MeV^{-1} under room temperature.[24,25] Though some organic-inorganic perovskites have demonstrated excellent performance in optoelectronic devices, the serous thermal quenching effects still restrict their applications of X-ray scintillators. $CH_3NH_3PbI_3$ and $CH_3NH_3PbBr_3$ exhibit a high light yield of ~150,000 photons MeV^{-1} at 10K, however, the high yields are measured as <1,000 photons MeV^{-1} under room temperature.[26] It should be noted that the thermal quenching effect is not limited to lead-containing perovskites. A light yield of ≈110,000 photons MeV^{-1} can be obtained for Rb_2AgBr_3, while the high yield dramatically decreases as the increased temperature.[27] Though the light yield of PEA_2MnCl_4 is expected to be 200,000 photons MeV^{-1} based on the theoretical calculations, the absence of X-ray RL signals was observed for PEA_2MnCl_4.[28] The same quenching phenomenon is also observed for PPA_2MnCl_4.[28] Up to know, the thermal quenching effect in perovskite-type X-ray scintillator is still under investigation and difficult to be predicted.

Taking all aspects into consideration, the advantages of the obtained HNTs@$Na_5Lu_9F_{32}$:Tb^{3+} are summarized in the following:

The good water-dispersibility and desirable compatibility with polymer matrices enable diverse aqueous processing approaches of radioluminescent foams, X-ray scintillating screens, and information encrypting hydrogels.

The large length-diameter ratios can improve the mechanical properties of HNTs@$Na_5Lu_9F_{32}$:Tb^{3+}-incorporated flexible X-ray scintillator screens.

The light yield of 12,300 photons MeV^{-1} can be obtained under room temperature, which is higher than well-known commercial scintillators including BaF_2, BGO, and GSO:Ce.

HNTs@$Na_5Lu_9F_{32}$:Tb^{3+} shows low cytotoxicity.

There is no need for harsh crystal growth conditions or high temperatures in synthesis and processing procedures.

The raw materials can be obtained and further processed at low cost.

HNTs@$Na_5Lu_9F_{32}$:Tb^{3+} exhibits good stability to heat and good light stability.

Supplementary Discussion 5: Investigation of the penetration abilities

A dried piece of 8.0 cm × 8.0 cm ×8.0 cm PUF was immersed in the HNTs@$Na_5Lu_9F_{32}$:Tb^{3+} aqueous dispersion (ca. 100 mg mL^{-1}) by fully compressing it three times and letting it soak for 3 min. The bottom was marked to distinguish it from other faces. The foam was wrung out in a mechanical roller and then dried under reduced pressure. The increased weight is calculated as 112%. Then the obtained composite foam was split in the middle, perpendicular to the bottom face. Five areas from bottom to up (0 cm, 2 cm, 4 cm, 6 cm, and 8 cm) were selected along the central axis in the cross-section and further analyzed by the SEM observation and elemental mapping studies (Supplementary Fig. 12).

The results indicate the distribution of Si, Al, Na, F, and Lu at the pore walls for all cases. Because of the relatively low content of Tb in the obtained HNTs@ $Na_5Lu_9F_{32}$:Tb^{3+}, the distribution of Tb is difficult to achieve. A similar phenomenon can also be found in Fig. 3m in the main text. Si, Al, and F are the three most abundant elements in the EDS results (Supplementary Table 3) and further quantitatively analyzed to evaluate the uniformity among the five areas from bottom to up (0 cm, 2 cm, 4 cm, 6 cm, and 8 cm). The Atom % of Al is detected as 15.82, 15.48, 14.79, 14.39, and 15.22%, respectively, with a relative standard deviation (RSD) of 3.72%. The Atom % of Si is measured as 20.62, 22.10, 21.33, 21.69, and 21.73 %, respectively, with an RSD of 2.60%. The Atom % of F is 44.44, 44.03, 46.41, 47.43, and 44.74%, respectively, with an RSD of 3.19%. All of the results suggest that the HNTs@ $Na_5Lu_9F_{32}$:Tb^{3+} shows a uniform distribution inside the 8.0 cm × 8.0 cm ×8.0 cm foam and a good penetration ability is demonstrated.

Supplementary Discussion 6: Different excitation–emission mechanisms

Organic materials composed of light atoms usually exhibit weak X-ray absorption.[29,30] On the other hand, X-ray beams hold much higher energy than UV/Vis light. A large number of triplet excitons can be generated with exposure to X-ray, which results in an intrinsic and huge loss channel.[31] Therefore, many conventional organic luminescent materials can only generate luminescence under UV light and cannot emit light under X-ray irradiation. Take 1,4-phenyldiboronic for an example, it can be used as a crosslinker with PVA to prepare UV luminescent hydrogels, which have been reported in our previous study,[2] while it cannot provide spectroscopic capabilities under X-ray excitation.

As for inorganic crystal scintillators, the excitation–emission mechanisms are also remarkably different between UV/Vis-induced PL and X-ray-induced RL processes. UV/Vis excitation process can directly excite the luminescent center, whereas the X-ray excitation process interacts with holes and electrons

from the host matrix.[32] The excitation that ultimately leads to RL should be due to an excitation of an electron from the valence band to the conduction band, while this process is difficult to take place in PL.[33,34] In the mechanism of PL, the excited valence electrons generated by optical excitation will return to the ground state, accompanied by emitting photons. The dopant ions can be directly excited by incident photon energy in this process. In contrast, in the RL case, the dopants are indirectly excited: X-ray irradiation first generates photoelectrons or Compton electrons; the energy carried by the electrons then excites the dopant ions. Consequently, energy loss may take place in the indirect RL process and give rise to different emission behaviors.[35] Some cases in prior reports have been summarized in Supplementary Table 4, which may be helpful for potential readers interested in this topic.

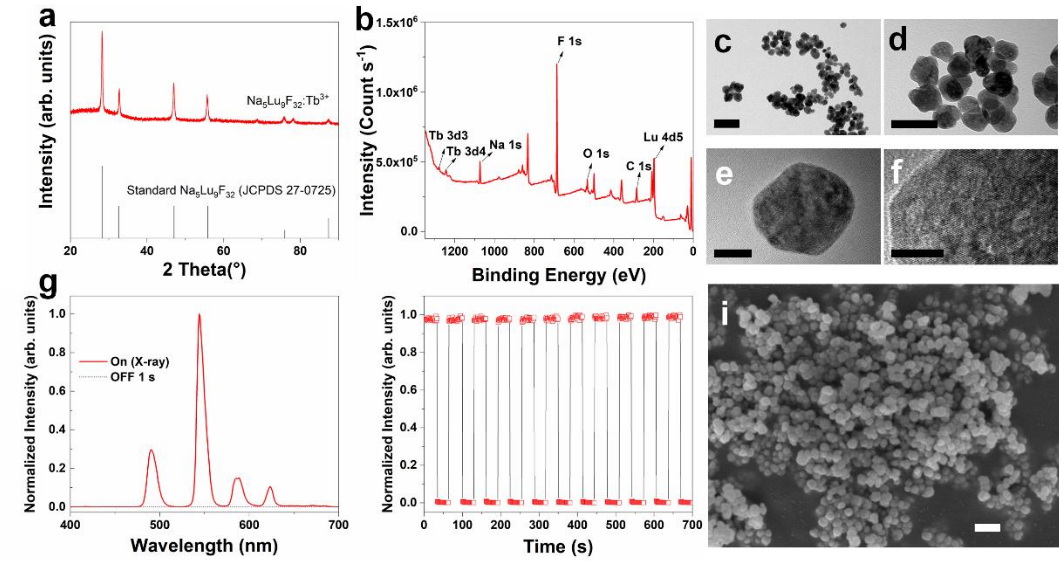

Supplementary Figure 1. Characterizations of Tb^{3+}-doped $Na_5Lu_9F_{32}$. a X-ray powder diffraction (XRD) pattern of Tb^{3+}-doped $Na_5Lu_9F_{32}$ ($Na_5Lu_9F_{32}$:Tb^{3+}) and the comparison with standard. **b** X-ray photoelectron spectroscopy (XPS) spectrum of $Na_5Lu_9F_{32}$:Tb^{3+}. **c** Transmission electron microscopy (TEM) image of $Na_5Lu_9F_{32}$:Tb^{3+} (bar: 200 nm). **d** TEM image of $Na_5Lu_9F_{32}$:Tb^{3+} (bar: 100 nm). **e** TEM image of $Na_5Lu_9F_{32}$:Tb^{3+} (bar: 20 nm). **f** TEM image of $Na_5Lu_9F_{32}$:Tb^{3+} (bar: 10 nm). **g** X-ray-excited radioluminescence (RL) spectrum of $Na_5Lu_9F_{32}$:Tb^{3+}. **h** In situ measurement of the luminescence intensity of $Na_5Lu_9F_{32}$:Tb^{3+} under X-ray with "On-Off" cycles. **i** Scanning electron microscope (SEM) image of $Na_5Lu_9F_{32}$:Tb^{3+} (bar: 200 nm).

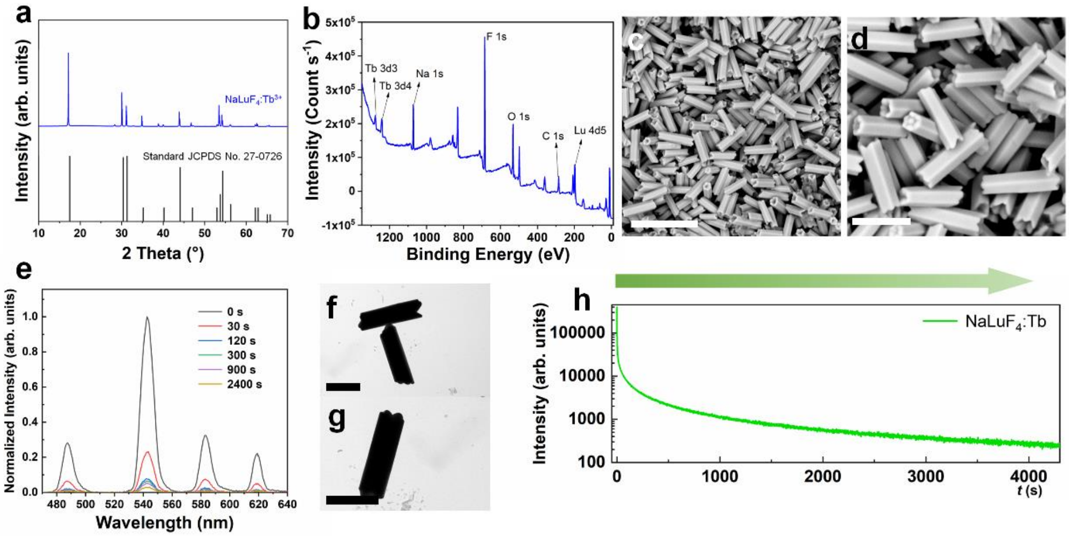

Supplementary Figure 2. Characterizations of Tb^{3+}-doped $NaLuF_4$. a X-ray powder diffraction (XRD) pattern of Tb^{3+}-doped $NaLuF_4$ ($NaLuF_4$:Tb^{3+}) and the comparison with standard. **b** X-ray photoelectron spectroscopy (XPS) spectrum of $NaLuF_4$:Tb^{3+}. **c** Scanning electron microscope (SEM) image of $NaLuF_4$:Tb^{3+} (bar: 8 μm). **d** SEM image of $NaLuF_4$:Tb^{3+} (bar: 3 μm). **e** X-ray-excited radioluminescence (RL) spectrum and afterglow spectra of $NaLuF_4$:Tb^{3+}. **f&g** Transmission electron microscopy (TEM) images of $NaLuF_4$:Tb^{3+} (bar: 2 μm). **h** Afterglow intensity from $NaLuF_4$:Tb^{3+} monitored at 544 nm as a function of time.

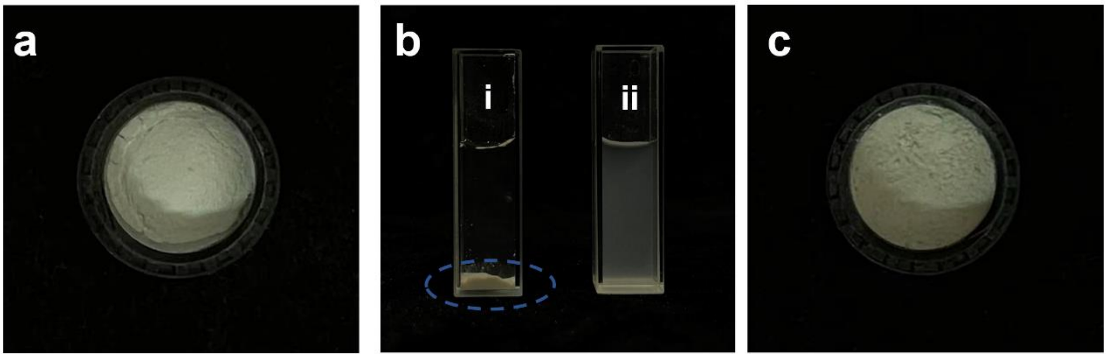

Supplementary Figure 3. Photographs of obtained scintillators. a Tb^{3+}-doped $Na_5Lu_9F_{32}$ ($Na_5Lu_9F_{32}:Tb^{3+}$) powder. **b** Water suspension of $Na_5Lu_9F_{32}:Tb^{3+}$ (**i**) and Tb^{3+}-doped $Na_5Lu_9F_{32}$ anchored halloysite nanotubes (HNTs@$Na_5Lu_9F_{32}:Tb^{3+}$) (**ii**). **c** HNTs@$Na_5Lu_9F_{32}:Tb^{3+}$ powder.

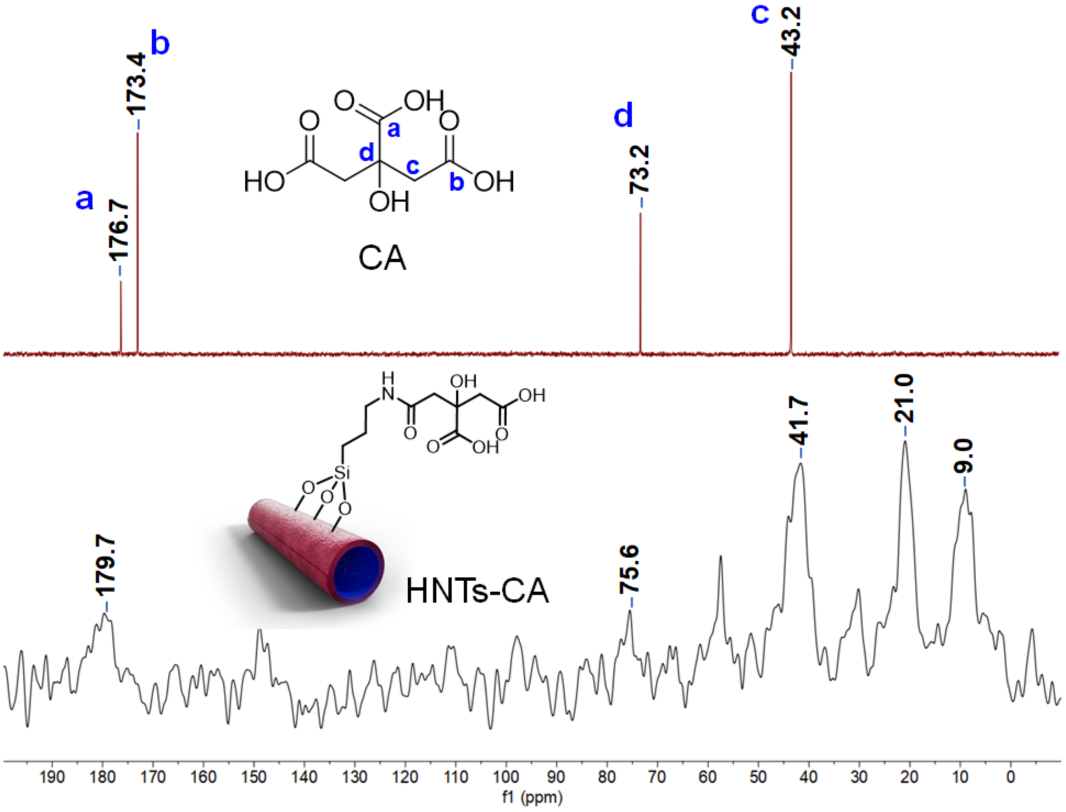

Supplementary Figure 4. Nuclear magnetic resonance results. Solid-state ^{13}C nuclear magnetic resonance (NMR) spectrum of citric acid-modified halloysite nanotubes (HNTs-CA) and ^{13}C NMR spectrum (DMSO-d_6) of citric acid (CA).

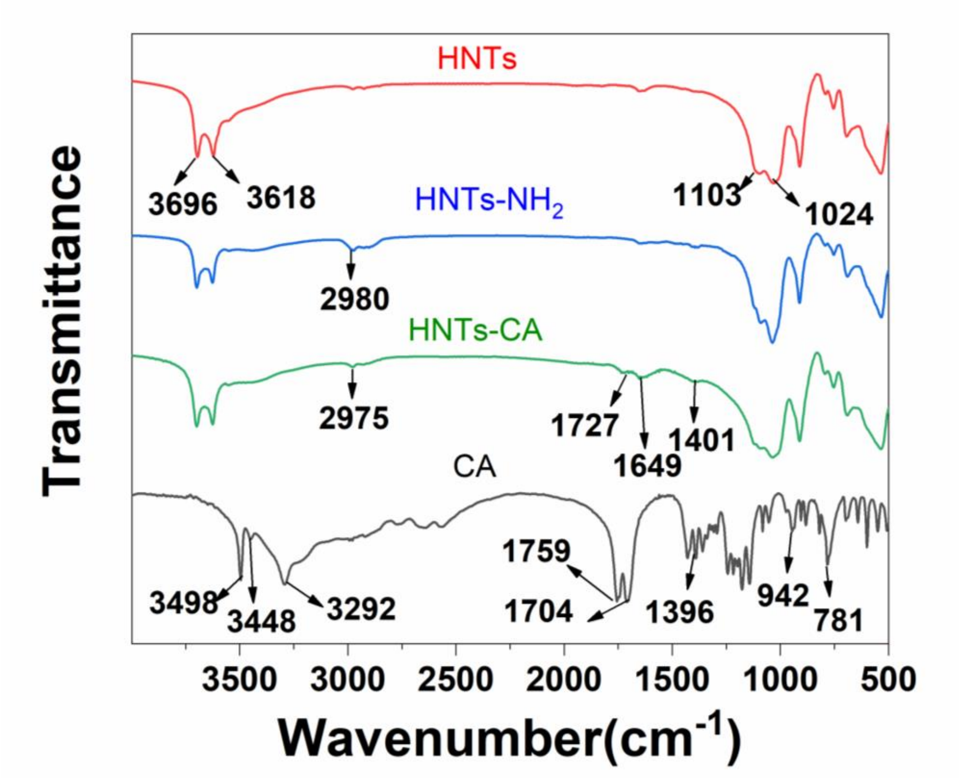

Supplementary Figure 5. Fourier transform infrared spectroscopy results. Fourier transform infrared spectroscopy (FTIR) spectra of halloysite nanotubes (HNTs), aminated halloysite nanotubes (HNTs-NH_2), citric acid-modified halloysite nanotubes (HNTs-CA), and citric acid (CA).

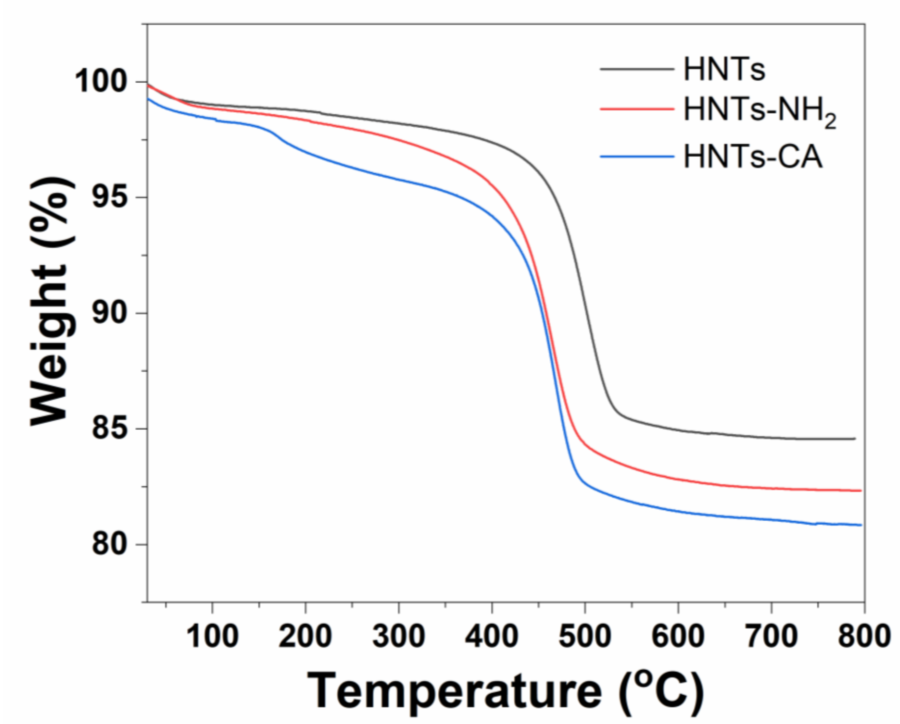

Supplementary Figure 6. Thermal gravimetric results. Thermal gravimetric analysis (TGA) curves of halloysite nanotubes (HNTs), aminated halloysite nanotubes (HNTs-NH_2), and citric acid-modified halloysite nanotubes (HNTs-CA).

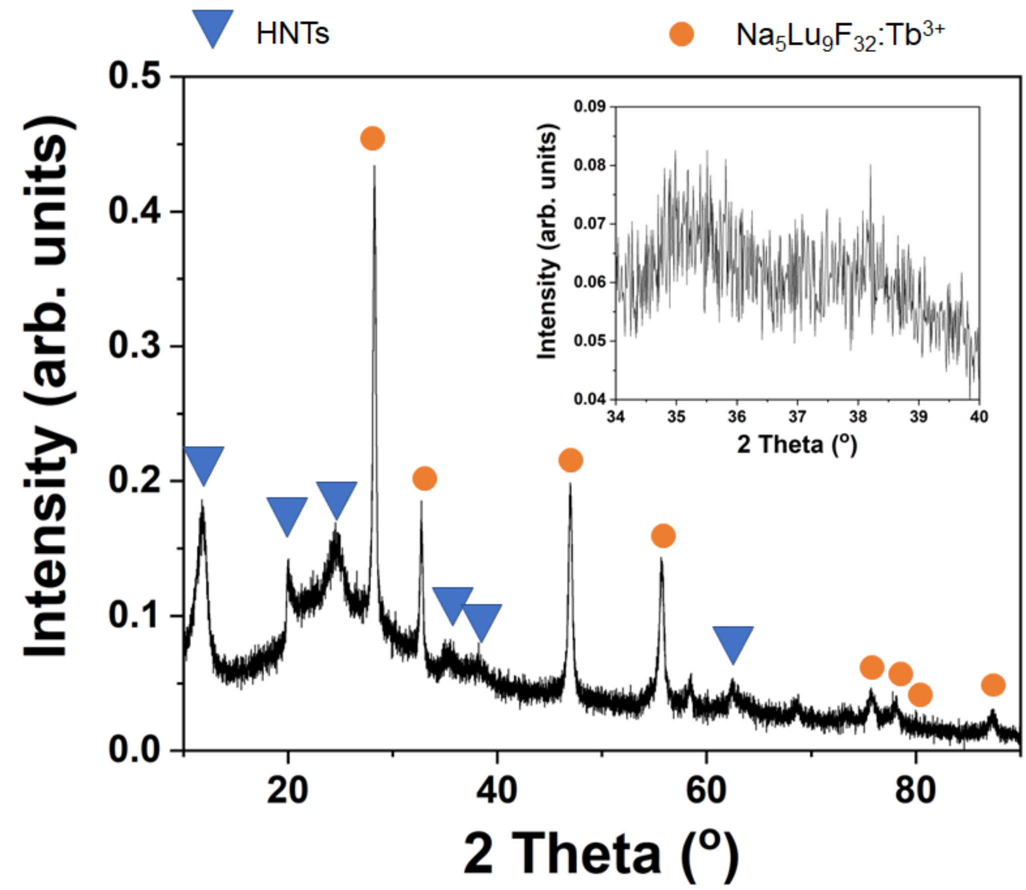

Supplementary Figure 7. X-ray powder diffraction results. X-ray powder diffraction (XRD) pattern of Tb^{3+}-doped $Na_5Lu_9F_{32}$ anchored halloysite nanotubes (HNTs@$Na_5Lu_9F_{32}$:Tb^{3+}) and the illustration of the affiliations (HNTs is abbreviated from halloysite nanotubes; $Na_5Lu_9F_{32}$:Tb^{3+} is abbreviated from Tb^{3+}-doped $Na_5Lu_9F_{32}$).

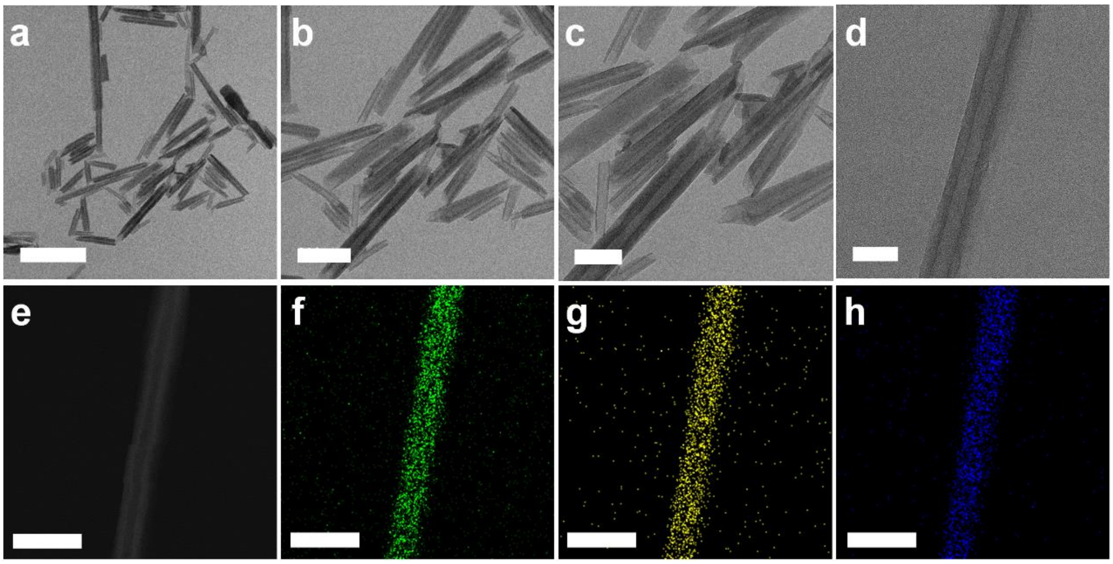

Supplementary Figure 8. Micromorphology characterizations of pristine halloysite nanotubes. a Transmission electron microscopy (TEM) image, bar: 500nm. **b** TEM image, bar: 200 nm. **c** TEM image, bar: 100 nm. **d** TEM image, bar: 50 nm. **e** High-angle annular dark-field (HAADF) scanning transmission electron microscopy (STEM) image bar: 100 nm. **f** Oxygen, bar: 100 nm. **g** Silicon, bar: 100 nm. **h** Aluminum, bar: 100 nm.

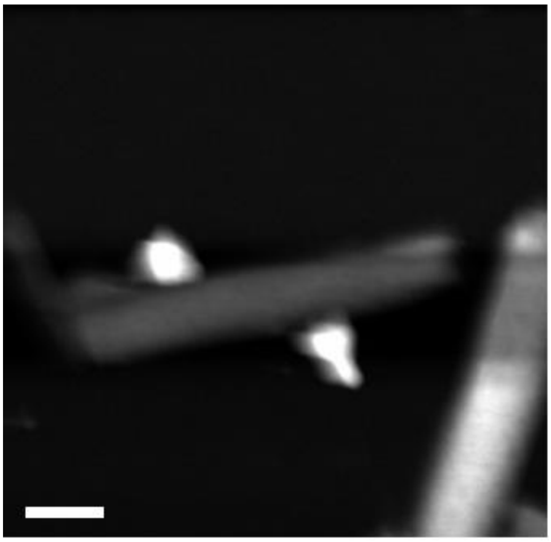

Supplementary Figure 9. High-angle annular dark-field scanning transmission electron microscopy image. bar: 100 nm

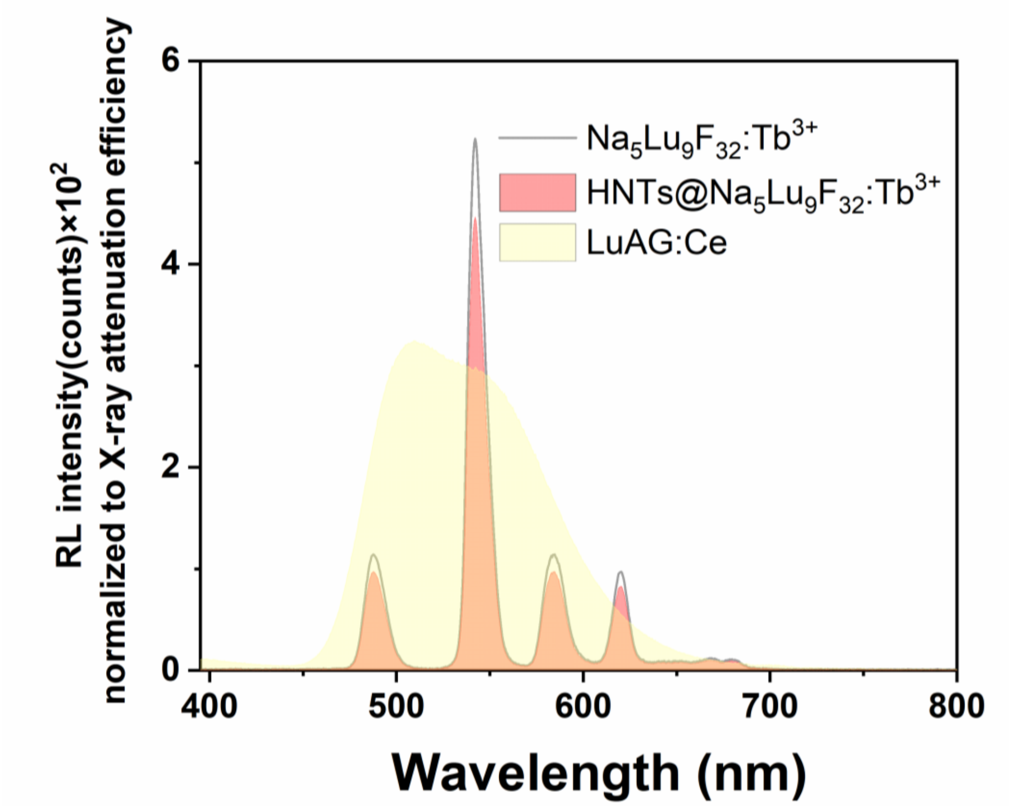

Supplementary Figure 10. Radioluminescence spectra for calculating light yields. RL is abbreviated from radioluminescence spectra; $Na_5Lu_9F_{32}:Tb^{3+}$ is abbreviated from Tb^{3+}-doped $Na_5Lu_9F_{32}$; $HNTs@Na_5Lu_9F_{32}:Tb^{3+}$ is abbreviated from Tb^{3+}-doped $Na_5Lu_9F_{32}$ anchored halloysite nanotubes.

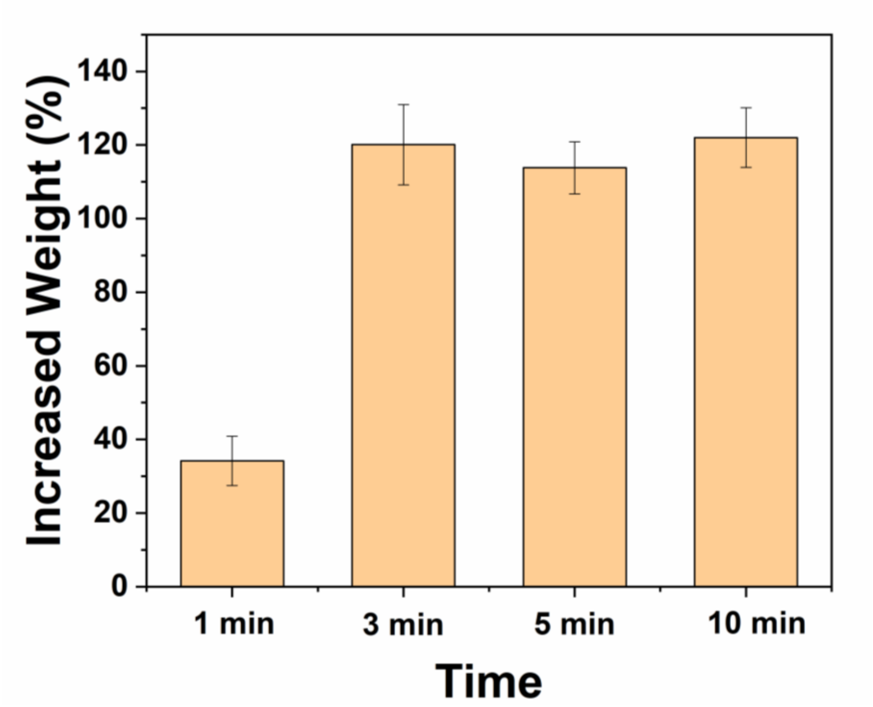

Supplementary Figure 11. Gravimetrical method results. Increased weight of the polyurethane foam (PUF) after soaking in Tb^{3+}-doped $Na_5Lu_9F_{32}$ anchored halloysite nanotubes (HNTs@$Na_5Lu_9F_{32}$:Tb^{3+}) solution in with different times.

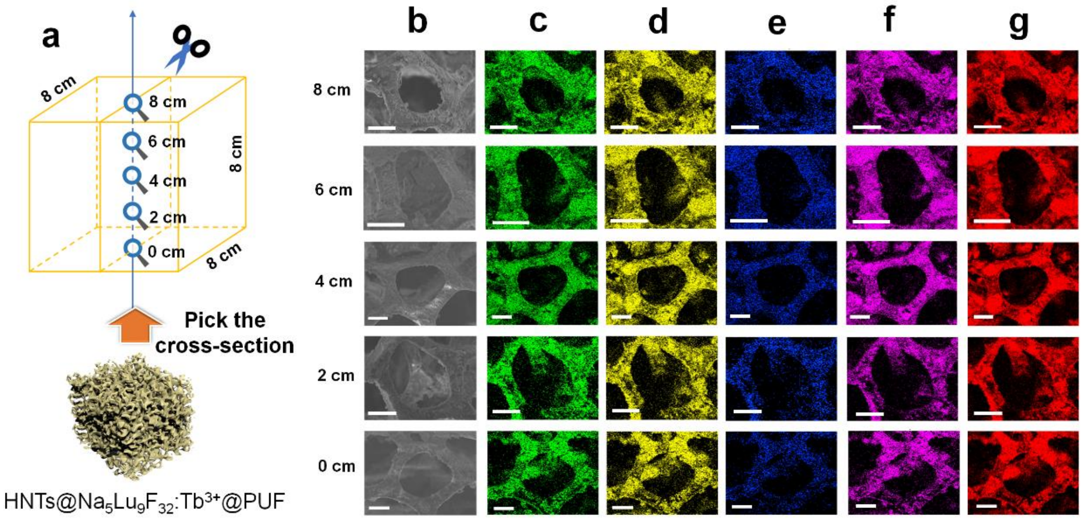

Supplementary Figure 12. Elemental analysis on the cross-section. a Illustration of the sampling methods of Tb^{3+}-doped $Na_5Lu_9F_{32}$ anchored halloysite nanotubes coated polyurethane foam (HNTs@$Na_5Lu_9F_{32}$:Tb^{3+}@PUF). **b** SEM images, bar: 100 μm. **c-g** Elemental mapping images (**c** Si, **d** Al, **e** Na, **f** F, and **g** Lu; bar: 100 μm).

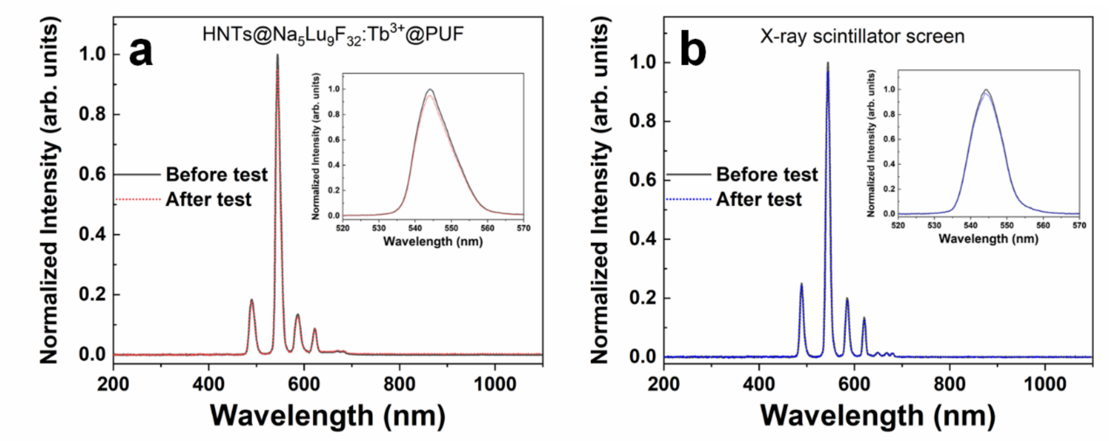

Supplementary Figure 13. Radioluminescence spectra in stability test. a Tb^{3+}-doped $Na_5Lu_9F_{32}$ anchored halloysite nanotubes coated polyurethane foam (HNTs@$Na_5Lu_9F_{32}$:Tb^{3+}@PUF). The inset image is the local enlarged image relating to the peak at 544 nm. **b** Tb^{3+}-doped $Na_5Lu_9F_{32}$ anchored halloysite nanotubes (HNTs@$Na_5Lu_9F_{32}$:Tb^{3+})-based rigid X-ray scintillator screen. The inset image is the local enlarged image relating to the peak at 544 nm.

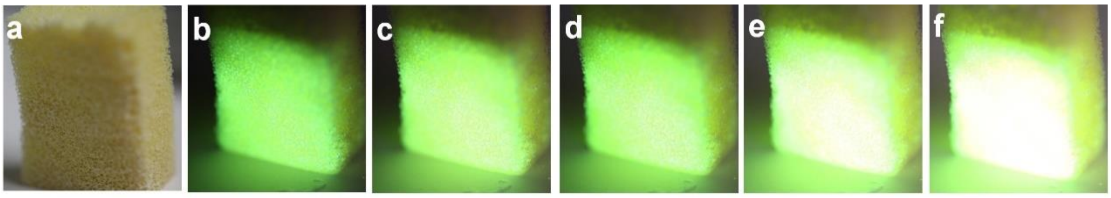

Supplementary Figure 14. Photographs of the obtained composite foams. a Tb^{3+}-doped $Na_5Lu_9F_{32}$ anchored halloysite nanotubes coated polyurethane foam (HNTs@$Na_5Lu_9F_{32}$:Tb^{3+}@PUF) taken under normal light. **b-f** HNTs@$Na_5Lu_9F_{32}$:Tb^{3+}@PUF taken under X-ray with different doses (3.1, 4.5, 5.0, 7.4, and 9.2 cGy s^{-1}).

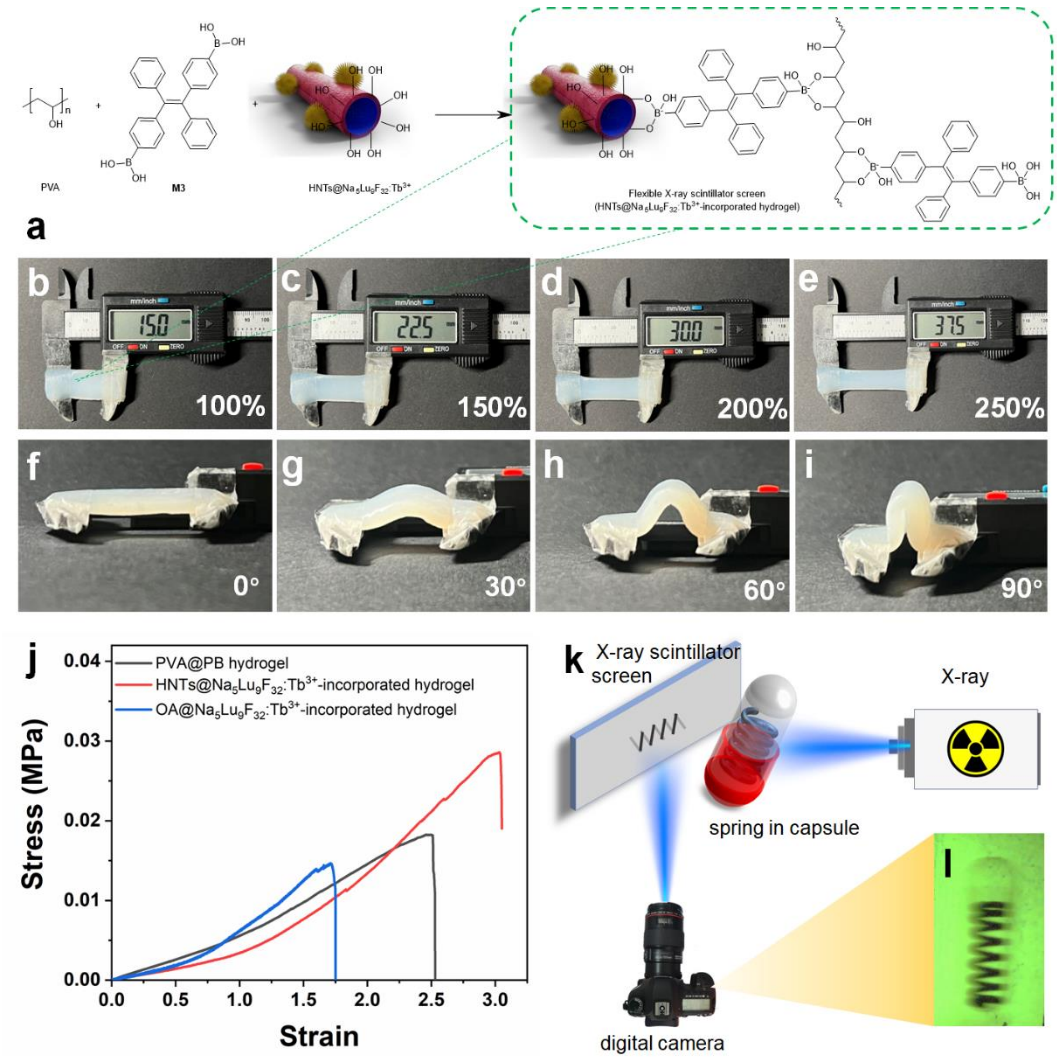

Supplementary Figure 15. Flexible X-ray scintillator screen. a Preparation of Tb^{3+}-doped $Na_5Lu_9F_{32}$ anchored halloysite nanotubes (HNTs@$Na_5Lu_9F_{32}$:Tb^{3+})-incorporated hydrogel which was used as flexible X-ray scintillator screen in this study. **b-e** Photographs of flexible X-ray scintillator screen at different stretch lengths. **f-i** Photographs of flexible X-ray scintillator screen under different bending angels. **j** Stress-strain curves of tensile testing. **k** Schematic diagram of the X-ray imaging system based on the as-prepared flexible X-ray scintillator screen. **l** The image relating to the capsule model taken from the digital camera.

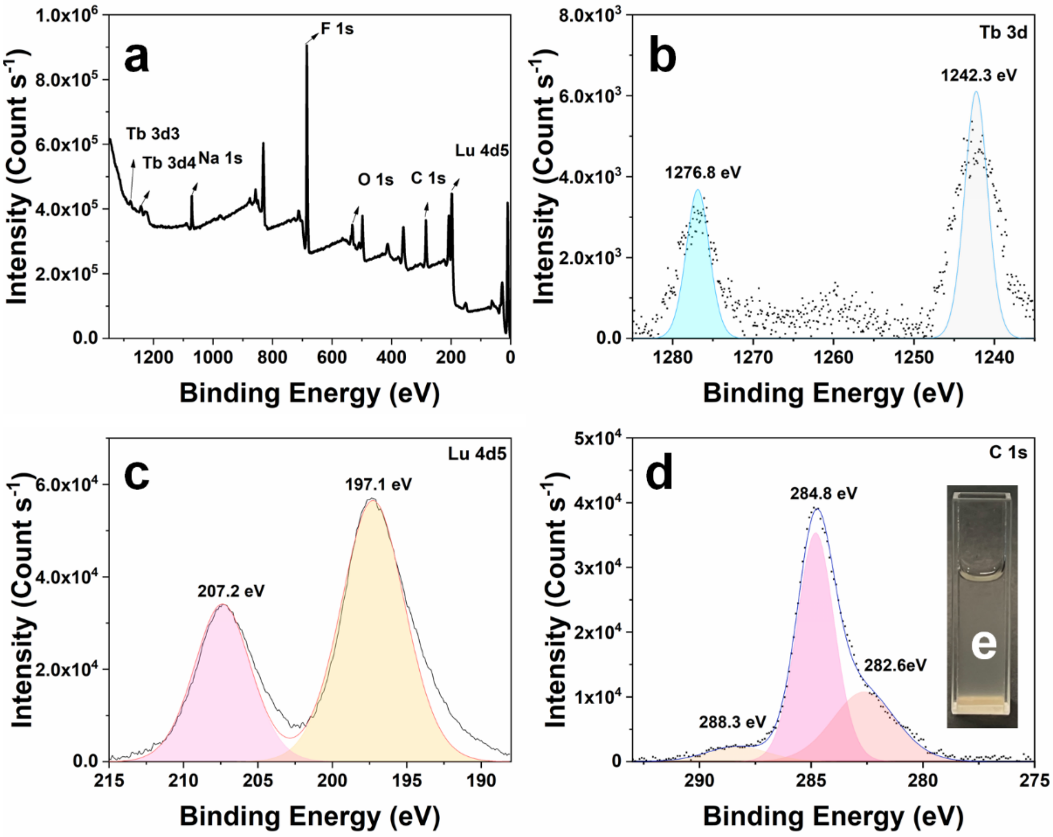

Supplementary Figure 16. Characterization of oleic acid-modified Tb^{3+}-doped $Na_5Lu_9F_{32}$. **a** X-ray photoelectron spectroscopy (XPS) pattern of oleic acid-modified Tb^{3+}-doped $Na_5Lu_9F_{32}$ (OA@$Na_5Lu_9F_{32}$:Tb^{3+}). **b** Tb 3d region. **c** Lu 4d5 region. **d** C 1s region. **e** Photograph of OA@$Na_5Lu_9F_{32}$:Tb^{3+} in water (1 mg mL^{-1}).

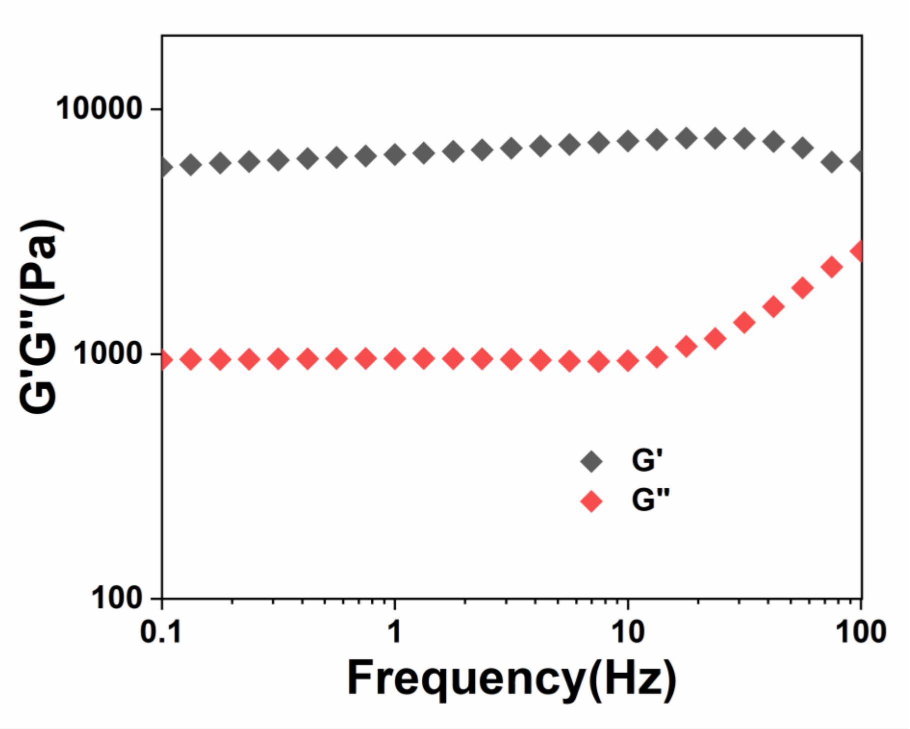

Supplementary Figure 17. Rheological sweep curves of the multi-layer hydrogel.

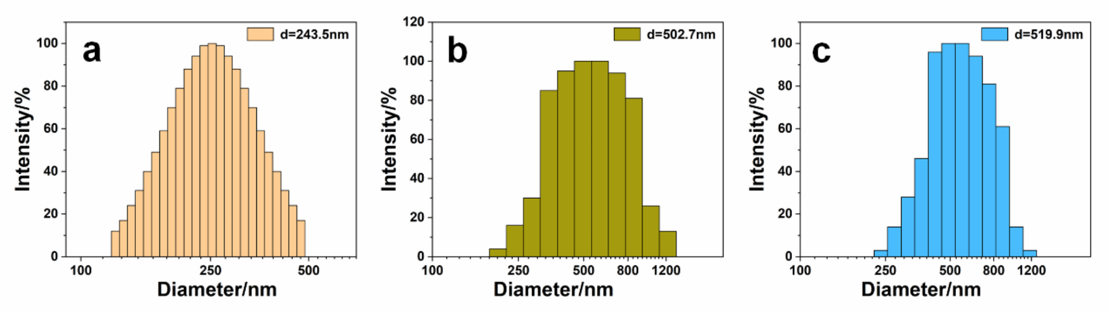

Supplementary Figure 18. Size distribution in aqueous solution. a halloysite nanotubes (HNTs): effective diameter: 244 nm; PDI: 0.10. **b** Tb^{3+}-doped $Na_5Lu_9F_{32}$ anchored halloysite nanotubes (HNTs@$Na_5Lu_9F_{32}$:Tb^{3+}): effective diameter: 503 nm; PDI: 0.15. **c** Tb^{3+}-doped $Na_5Lu_9F_{32}$ ($Na_5Lu_9F_{32}$:Tb^{3+}): effective diameter: 520 nm; PDI: 0.083.

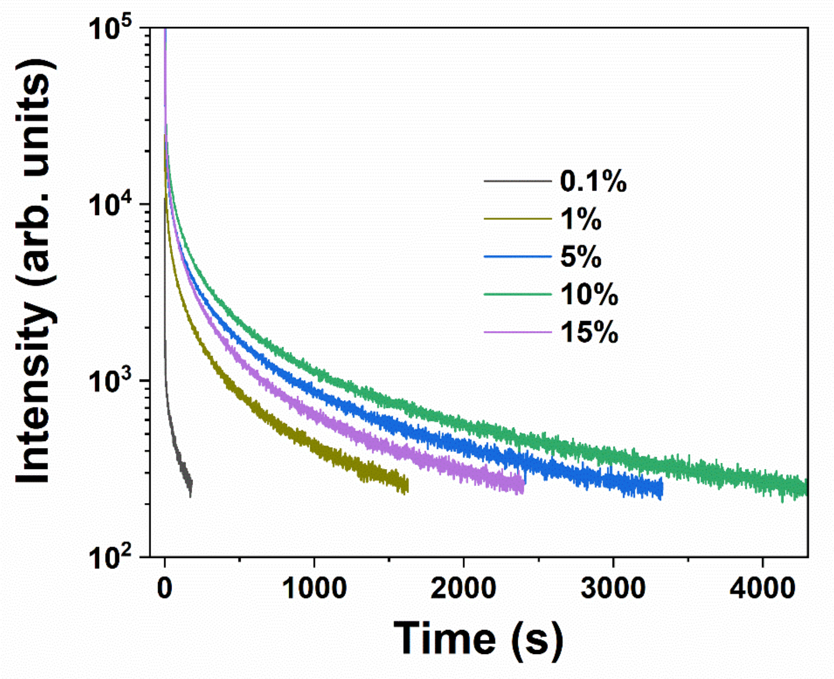

Supplementary Figure 19. Afterglow results. Afterglow intensity from Tb^{3+}-doped $NaLuF_4$ ($NaLuF_4:Tb^{3+}$) with different doping concentration of Tb^{3+} monitored at 544 nm as a function of time.

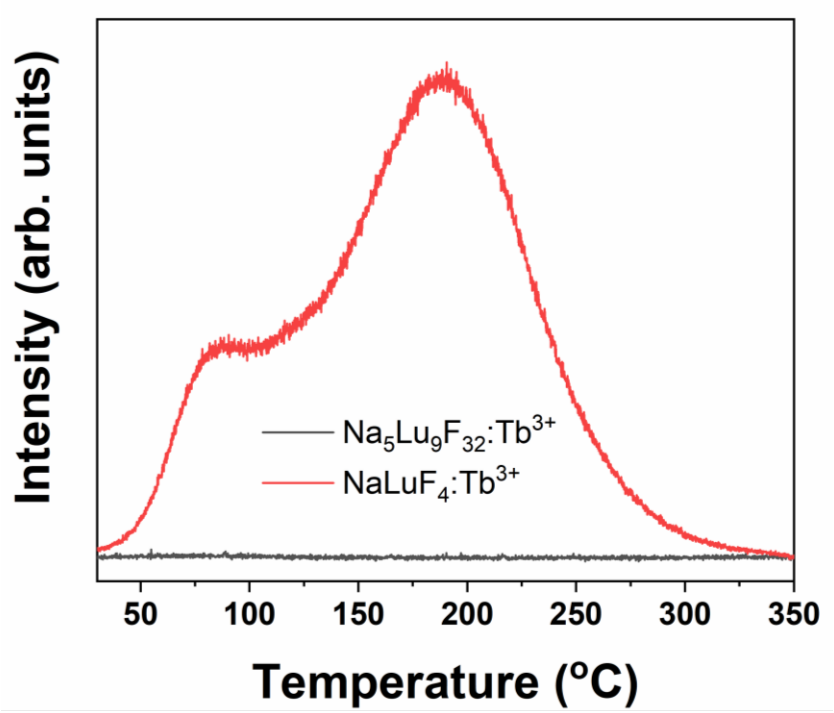

Supplementary Figure 20. Thermoluminescence spectra.

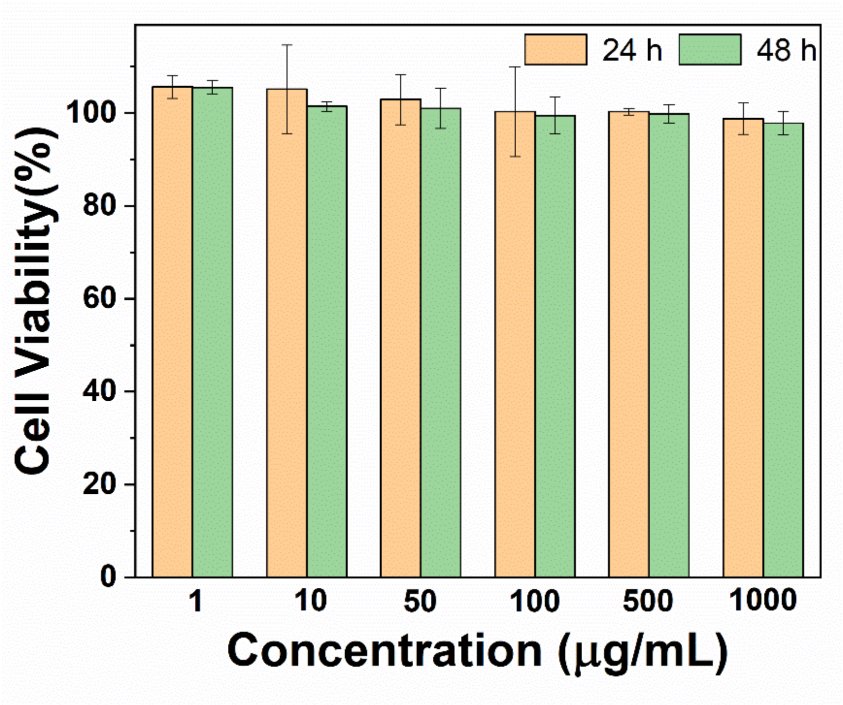

Supplementary Figure 21. Cell viability data.

Supplementary Table 1. Comparisons between the obtained $Na_5Lu_9F_{32}:Tb^{3+}$-anchored halloysite nanotubes ($HNTs@Na_5Lu_9F_{32}:Tb^{3+}$) with non-perovskite-type X-ray scintillators that have been reported in literature.

Sample	λ_{em}	Light yield (photons MeV^{-1})	Application	Material type	Processing method
$Al(PO_3)_3$–$CsPO_3$–CsBr–$CeBr_3$ glass[36]	360 nm	2,700	X-ray detection	Glass	Melted at 950 ºC
BaF_2 crystals[37,38]	350 and 425 nm	1,400	Radiation absorption	Polystyrene composite film	Evaporation ($C_2H_4Cl_2$/CCl_4)
BaF_2:Y crystals[39]	300 nm	2,000	X-ray imaging	/	/
$Bi_4Ge_3O_{12}$ (BGO) crystals[40,41]	480 nm	8,500	X-ray detector	Hard film	Rapid annealing (750~800ºC)
$CsGd_2F_7$: Ce nano-glass[42]	380 nm	827	X-ray imaging	Hard film	Heat at 710 or 720 ºC
Cu_4I_4: Tb MOFs[43]	494, 547, 587, and 622 nm	29,379	X-ray imaging	Flexible film	Evaporation (chloroform)
Gd_2SiO_5 (GSO):Ce crystals[44,45]	450 nm	7,000	X-ray imaging	Hard film	Annealing (400~1,000ºC)
$LaBr_3$:Ce single crystal[46]	380 nm	60,000	Imager and spectrometer	Single crystal	Bridgman systems[47]
LuF_3:Nd crystals[48]	180, 230, and 250 nm	1,200	Ultraviolet light emitting	Thin film	Laser deposition at 400 ºC
$Lu_2Si_2O_7$:Nd single crystal[49]	800–900 nm	800	/	/	/
$Lu_2Si_2O_7$:Pr single crystal[50]	300–340 nm	9,700	/	/	/
LYSO:Ce single crystal[51]	420 nm	29,000	X-ray imaging	Single crystal	Czochralski method
$K_3Gd(PO_4)_2$:Ce crystals[52]	335 and 360 nm	10,217	X-ray detection	Crystals	Calcined at 1,223 K
$KLaF_4:Ce@KLa_{1-x}Gd_xF_4$ composite[53]	380 nm	853	X-ray dosimetry	Fiber	Heating at 650 ºC
PVK:Bi composite[54]	440 nm	5,600	X-ray detection	Plastic piece	Evaporation (THF)
$Sr_3NbGa_3Si_2O_{14}$ crystals[55]	400 nm	850	/	/	/
$Y_3Al_5O_{12}$ (YAG):Ce single crystal[51]	550 nm	17,000	X-ray imaging	Single crystal	Czochralski method
ZnO single crystal[56,57]	390 nm	9,000	X-ray imaging	Nanowire field emitter	Electron beam evaporation (500ºC)
ZnO:Ga single crystal[58]	389 nm	714	Diagnose the distribution of cathode electron emission	Single crystal	Hydro-thermal method
$Na_5Lu_9F_{32}:Tb^{3+}$ crystals*	489, 544, 585, and 620 nm	15,800	/	/	/
$HNTs@Na_5Lu_9F_{32}:Tb^{3+}$ nanotubes*	489, 544, 585, and 620 nm	12,300	X-ray detection	PUF composite foam	Assembling in water under RT[a]
			X-ray imaging	Hard film	Evaporation (water)
			X-ray imaging	Flexible film	In situ crosslinking in water
			Information encryption	Hydrogel	In situ crosslinking in water

* Reported in this study

[a] "RT" is abbreviated from room temperature

Supplementary Table 2. Comparisons between the obtained $Na_5Lu_9F_{32}:Tb^{3+}$-anchored halloysite nanotubes (HNTs@$Na_5Lu_9F_{32}:Tb^{3+}$) with perovskite-based X-ray scintillators that have been reported in literature.

Sample	λ_{em}	Light yield (photons MeV^{-1})	Application	Material type	Processing method
$CaHfO_3:Ce$ single crystal[59]	430 nm	7,800	/	/	/
$Cs_2BaBr_4:Tl$ crystals[60]	380 and 510 nm	1,700~2,700	/	/	/
Cs_3BiCl_6 crystals[61]	390 and 600~700 nm	800	/	/	/
$Cs_2HfCl_6:Te$ crystals[62]	575 nm	9,000~13,100	/	/	/
$CsCaCl_3$ crystals[63]	250 and 305 nm	410	/	/	/
$CsCaCl_3:Ce$ crystals[63]	375 nm	7,600	/	/	/
$Cs_3Cu_2I_5$ crystals[64]	570 nm	31,700	X-ray imaging	Hard film	390 °C heating in nitrogen-filled glovebox
$Cs_3Cu_2I_5$ nanocrystals[65]	445 nm	79,279	X-ray imaging	Thick film	Evaporation (hexane)
$CsPbBr_3$ crystals[24,66]	533, 548, and 573 nm	50,000 (7 K[a])[24] < 500 (RT[b])[25]	X-ray detection	Flexible film	Evaporation (dichloromethane)
$CsPbBr_3$ nanosheets[67]	525 nm	21,000	X-ray imaging	Hard film	Assembly in toluene
$CsPbBr_3:Eu$ quantum dots[68]	595, 616, 654, and 701 nm	10,100	X-ray imaging	Glass-ceramic	500 °C heating
$CsPbBr_3:F$ nanocrystals[69]	/	8,500	X-ray detection	Flexible film	Evaporation (dichloromethane)
$CsPbBr_3:Lu$ nanocrystals[70]	516 nm	/	X-ray imaging	Hard film	Melt (1,200 °C)-annealed (420 °C)-heat (500 °C)
$CsPbBr_3$@BaF_2 composite[71]	Tunable from 435 to 648 nm	6,300	X-ray detection	Hard screen	Evaporation (dodecane/ethyl acetate)
$CsPbCl_3$ single crystal[25]	415 nm	330	/	/	/
$Cs_2SnF_6:Mn$ nanocrystals[72]	600-650 nm	3,000	X-ray imaging	Flexible film	Evaporation (dichloromethane)
$CsSrCl_3:Ce$ crystals[73]	350-400 nm	8,600	/	/	/
$Cs_2ZnBr_4:Mn$ crystals[74]	526 nm	15,600	X-ray imaging	Flexible film	Evaporation (ethanol)
Cs_2ZnCl_4 nanorod[75]	254, 305, 400, 500, and 760 nm	100-300	/	/	/
Rb_2AgBr_3 crystals[27]	480 nm	110,000 (80K)	X-ray imaging	Thick film	550 °C heating
$TlCdCl_3$ crystal[76]	450 nm	2,200	/	/	/
$(A)_2(MA)_{n-1}Pb_nBr_{3n+1}$ nanocrystals[77]	542 nm	/	X-ray imaging	Flexible film	Spin-coating using chlorobenzene in nitrogen-filled glovebox
$(BM)_2PbBr_4$ crystals[78]	480 nm	3,190	/	/	/
$(CH_3NH_3)PbI_3$ crystals[26]	770 nm	150,000 (10 K) < 1,000 (RT)	/	/	/
$(CH_3NH_3)PbBr_3$ crystals[26]	540 nm	150,000 (10 K) < 1,000 (RT)	/	/	/
$(C_6H_5C_2H_4NH_3)_2PbBr_4$ single crystal[79]	410 nm	14,000	Radioactive element detection	Single crystal	Evaporation (DMF)
$(C_6H_5C_2H_4NH_3)_2PbI_4$ single crystal[80]	560	2,900	/	/	/

$(C_{13}H_{14}N_3)_3SbCl_6$ crystals[81]	/	2,000	/	/	/
$((C_{38}H_{34}P_2)MnBr_4$ single crystal[82]	517 nm	~ 80,000	X-ray imaging	Flexible film	Grind into powder and dispersed in Organosilicone
$(C_{24}H_{20}P)_2MnBr_4$ nanocrystals[83]	520 nm	/	X-ray imaging	Flexible film	Evaporation (DMF)
$(EDBE)PbCl_4$ crystals[26]	520 nm	1,200,000 (130 K)<br>9000 (RT)	/	/	/
$(PEA)_2MnCl_4$ crystals[28]	604 nm	200,000 (DFT[c])<br>~0 (RT)	/	/	/
$(PEA)_2PbBr_4$: Li single crystal[84]	450 to 750 nm	11,000	X-ray imaging	Hard film	Evaporation (DMSO)
$(PEA)_2PbI_4$ crystals[85]	532 and 660 nm	10,000 (10 K)<br>1,000 (RT)	/	/	/
$(S\text{-}3AP)PbBr_3Cl \cdot H_2O$ single crystal[86]	575 nm	19,000	X-ray imaging	Single crystal	Grow from HCl&HBr
$Na_5Lu_9F_{32}:Tb^{3+}$ crystals*	489, 544, 585, and 620 nm	15,800 (RT)	/	/	/
$HNTs@Na_5Lu_9F_{32}:Tb^{3+}$ nanotubes*	489, 544, 585, and 620 nm	12,300 (RT)	X-ray detection	PUF composite foam	Assembling in water under RT
			X-ray imaging	Hard film	Evaporation (water)
			X-ray imaging	Flexible film	In situ crosslinking in water
			Information encryption	Hydrogel	In situ crosslinking in water

* Reported in this study;

[a] "K" is abbreviated from Kelvin;

[b] "RT" is abbreviated from room temperature;

[c] "DFT" is abbreviated from density functional theory.

Supplementary Table 3. Summary and analysis of the atom percentage of Al, Si, and F in the cross-section of $Na_5Lu_9F_{32}$:Tb^{3+}-anchored halloysite nanotubes coated polyurethane foam (HNTs@$Na_5Lu_9F_{32}$:Tb^{3+}@PUF) (8.0 cm × 8.0 cm ×8.0 cm) from SEM-EDS

	Atom % - Al	Atom % - Si	Atom % - F
0 cm	15.82	20.62	44.44
2 cm	15.48	22.10	44.03
4 cm	14.79	21.33	46.41
6 cm	14.39	21.69	47.43
8 cm	15.22	21.73	44.74
Average	15.14	21.49	45.41
SD	0.56	0.56	1.45
RSD	3.72	2.60	3.19

Supplementary Table 4. Summary of the materials exhibit different emission behaviors between UV/Vis-induced photoluminescence and X-ray-induced radioluminescence.

Sample	UV/Vis-induced photoluminescence	X-ray-induced radioluminescence
LaB_3O_6:Ce crystals[87]	λ_{em} = 303 nm (λ_{ex} = 277 nm)	λ_{em} = 323 nm
Y_3TaO_7:Tm crystals[88]	λ_{em} = 460, 471, 487, 610, 632, and 655 nm	λ_{em} = 355, 392, 455, 472, 487, 515, 658, and 797 nm
$NaMgF_3$:Sm nanoparticles[89]	λ_{em} = 450 (broad), 557, 593, 611, 639, and 698 nm (λ_{ex} = 280 nm)	λ_{em} = 555, 599, 636, 694, 723, and 800 nm
CaF_2:Th crystals[90]	Distinct peaks between 260 and 500 nm	λ_{em} = 230 and 400 nm
$KMgF_3$:Eu nanoparticles[91]	λ_{em} = 360 nm (λ_{ex} = 290 nm)	λ_{em} = 540–640 nm
$La_2Hf_2O_7$: Eu nanoparticles[92]	λ_{em} = 582, 612, 630, and 712 nm (λ_{ex} = 258 nm; λ_{max} locates at 612 or 630 nm)	Multi-peaks in the region 580–630 nm (λ_{max} occurs before 600 nm) and 700-725 nm
Cs_3BiCl_6 crystals[61]	λ_{em} = 390 nm (λ_{ex} = 325 nm)	In addition to the luminescence band at 390 nm, another band was observed at 600-700 nm
$CdTa_2O_6$:Eu crystals[32]	λ_{em} = 592, 612, and 623 nm (λ_{ex} = 465.7 nm)	λ_{em} = 430, 592, 599, 619, 662, 713, 754, and 822 nm
Yb^{2+}-doped silica glass[35]	λ_{em} = 506 nm (λ_{ex} = 399 nm)	λ_{em} = 525 nm
$BaSnO_3$: Tb ceramic[93]	λ_{em} = 541 and 897 nm (λ_{ex} =325 nm)	λ_{em} = 492, 541, 583, 619, and 897 nm
$NaMgF_3$: Mn nanoparticles[94]	λ_{em} = 498 and 602 nm (λ_{ex} =396 nm)	λ_{em} = 602 nm
$(PEA)_2MnCl_4$ crystals[28]	λ_{em} = 604 nm (λ_{ex} =366 nm)	Non-emission at room temperature
$(PPA)_2MnCl_4$ crystals[28]	λ_{em} = 581 nm (λ_{ex} =366 nm)	Non-emission at room temperature

Supplementary References

1 Su, Z. *et al.* Coumarin-anchored halloysite nanotubes for highly selective detection and removal of Zn(II). *Chem. Eng. J.* **393**, 124695 (2020).

2 Zhang, B., Li, S., Wang, Y., Wu, Y. & Zhang, H. Halloysite nanotube-based self-healing fluorescence hydrogels in fabricating 3D cube containing UV-sensitive QR code information. *J. Colloid Interface Sci.* **617**, 353-362 (2022).

3 Feng, Z. *et al.* In-situ repair of marine coatings by a Fe_3O_4 nanoparticle-modified epoxy resin under seawater. *Chem. Eng. J.* **430**, 132827 (2022).

4 Cheng, Y. *et al.* Enhancing light yield of Tb^{3+}-doped fluoride nanoscintillator with restricted positive hysteresis for low-dose high-resolution X-ray imaging. *Nano Res.* **16**, 3339−3347 (2023).

5 Midgley, S. Energy resolution for accurate measurements of the X-ray linear attenuation coefficient. *Radiat. Phys. Chem.* **75**, 936-944 (2006).

6 Yah, W. O. *et al.* Biomimetic dopamine derivative for selective polymer modification of halloysite nanotube lumen. *J. Am. Chem. Soc.* **134**, 12134-12137 (2012).

7 Li, Y., Gecevicius, M. & Qiu, J. Long persistent phosphors—from fundamentals to applications. *Chem. Soc. Rev.* **45**, 2090-2136 (2016).

8 Xu, J. & Tanabe, S. Persistent luminescence instead of phosphorescence: history, mechanism, and perspective. *J. Lumin.* **205**, 581-620 (2019).

9 Lyu, T. & Dorenbos, P. Bi^{3+} acting both as an electron and as a hole trap in La-, Y-, and $LuPO_4$. *J. Mater. Chem. C* **6**, 6240-6249 (2018).

10 Pei, P. *et al.* X-ray-activated persistent luminescence nanomaterials for NIR-II imaging. *Nat. Nanotech.* **16**, 1011-1018 (2021).

11 Hu, Y. *et al.* X-ray-excited super-long green persistent luminescence from Tb^{3+} monodoped β-$NaYF_4$. *J. Phys. Chem. C* **124**, 24940-24948 (2020).

12 Dorenbos, P. Mechanism of persistent luminescence in Eu^{2+} and Dy^{3+} codoped aluminate and silicate compounds. *J. Electrochem. Soc.* **152**, H107 (2005).

13 Dorenbos, P. Mechanism of persistent luminescence in $Sr_2MgSi_2O_7$: Eu^{2+}; Dy^{3+}. *Phys. Status. Solidi B Basic. Res.* **242**, R7-R9 (2005).

14 Li, L. *et al.* Mechanism of the trivalent lanthanides' persistent luminescence in wide bandgap materials. *Light Sci. Appl.* **11**, 51 (2022).

15 Evans, D. *et al.* Determination of the optical band-gap energy of cubic and hexagonal boron nitride using luminescence excitation spectroscopy. *J. Phys. Condens. Matter* **20**, 075233 (2008).

16 Muhammad, Z. *et al.* Revealing the quasiparticle electronic and excitonic nature in cubic, tetragonal, and hexagonal phases of $FAPbI_3$. *AIP Adv.* **12**, 025330 (2022).

17 Simon, J. *et al.* Direct comparison of recombination dynamics in cubic and hexagonal GaN/AlN quantum dots. *Phys. Rev. B* **68**, 035312 (2003).

18 Sudheendra, L. *et al.* $NaGdF_4$:Eu^{3+} Nanoparticles for Enhanced X-ray Excited Optical Imaging. *Chem. Mater.* **26**, 1881-1888 (2014).

19 Liu, X. *et al.* Binary temporal upconversion codes of Mn^{2+}-activated nanoparticles for multilevel anti-counterfeiting. *Nat. Commun.* **8**, 899 (2017).

20 Tang, Z. *et al.* Highly efficient and ultralong afterglow emission with anti-thermal quenching from $CsCdCl_3$: Mn perovskite single crystals. *Angew. Chem. Int. Ed.* **61**,

e202210975 (2022).
21 Guo, H. *et al.* Cyan emissive super-persistent luminescence and thermoluminescence in $BaZrSi_3O_9:Eu^{2+}$, Pr^{3+} phosphors. *J. Mater. Chem. C* **5**, 2844-2851 (2017).
22 Van den Eeckhout, K., Bos, A. J., Poelman, D. & Smet, P. F. Revealing trap depth distributions in persistent phosphors. *Phys. Rev. B* **87**, 045126 (2013).
23 Du, J., Feng, A. & Poelman, D. Temperature dependency of trap‐controlled persistent luminescence. *Laser Photonics Rev.* **14**, 2000060 (2020).
24 Mykhaylyk, V. B. *et al.* Bright and fast scintillations of an inorganic halide perovskite $CsPbBr_3$ crystal at cryogenic temperatures. *Sci. Rep.* **10**, 8601 (2020).
25 Kobayashi, M. *et al.* Scintillation characteristics of $CsPbCl_3$ single crystals. *Nucl. Instrum. Methods. Phys. Res. B* **592**, 369-373 (2008).
26 Birowosuto, M. D. *et al.* X-ray scintillation in lead halide perovskite crystals. *Sci. Rep.* **6**, 37254 (2016).
27 Zhang, M. Y. *et al.* Metal halide scintillators with fast and self-absorption-free defect-bound excitonic radioluminescence for dynamic X-ray Imaging. *Adv. Funct. Mater.* **31**, 2007921 (2021).
28 Hardhienata, H. *et al.* Optical and X-ray scintillation properties of X_2MnCl_4 (X = PEA, PPA) perovskite crystals. *J. Phys. D Appl. Phys.* **53**, 455303 (2020).
29 Hajagos, T. J., Liu, C., Cherepy, N. J. & Pei, Q. High‐Z sensitized plastic scintillators: a review. *Adv. Mater.* **30**, 1706956 (2018).
30 Wang, X. *et al.* Organic phosphors with bright triplet excitons for efficient X-ray-excited luminescence. *Nat. Photonics* **15**, 187-192 (2021).
31 Ma, W. *et al.* Thermally activated delayed fluorescence (TADF) organic molecules for efficient X-ray scintillation and imaging. *Nat. Mater.* **21**, 210-216 (2022).
32 İlhan, M. & Keskin, İ. Ç. Photoluminescence, radioluminescence and thermoluminescence properties of Eu^{3+} doped cadmium tantalate phosphor. *Dalton T.* **47**, 13939-13948 (2018).
33 Schuyt, J. J. & Williams, G. V. M. Quenching of the Sm^{2+} luminescence in $NaMgF_3$:Sm via photothermal ionization: alternative method to determine divalent lanthanide trap depths. *Appl. Phys. Lett.* **115**, 181104 (2019).
34 Chen, Q. *et al.* All-inorganic perovskite nanocrystal scintillators. *Nature* **561**, 88-93 (2018).
35 Liu, S. *et al.* Photoluminescence and radioluminescence properties of Yb^{2+}-doped silica glass. *Mater. Lett.* **144**, 43-45 (2015).
36 Nakabayashi, Y., Fujimoto, Y., Koshimizu, M. & Asai, K. Photoluminescence and scintillation properties of $Al(PO_3)_3$–$CsPO_3$–CsBr–$CeBr_3$ glass scintillators. *J. Mater. Sci. Mater. Electron.* **33**, 19846-19853 (2022).
37 Ota, R. & Uenoyama, S. Plasmonic ultraviolet filter for fast-timing applications. *Nanophotonics* **12**, 743-752 (2023).
38 Demkiv, T. M., Halyatkin, O. O., Vistovskyy, V. V., Gektin, A. V. & Voloshinovskii, A. S. Luminescent and kinetic properties of the polystyrene composites based on BaF_2 nanoparticles. *Nucl. Instrum. Methods. Phys. Res. A* **810**, 1-5 (2016).
39 Hu, C. *et al.* BaF_2: Y and ZnO: Ga crystal scintillators for GHz hard X-ray imaging.

Nucl. Instrum. Methods. Phys. Res. A **950**, 162767 (2020).
40 Chewpraditkul, W. *et al.* Luminescence and scintillation of Ce^{3+} - doped oxide glass with high Gd_2O_3 concentration. *Phys. Status Solidi A* **208**, 2830-2832 (2011).
41 Duan, M. *et al.* Deposition of scintillating layers of bismuth germanate (BGO) films for X-ray detector applications. *IEEE Nucl. Sci. Symp. Conf. Rec.* **1**, 845-847 (1997).
42 Li, W. *et al.* Intense radioluminescence from transparent $CsGd_2F_7$: Ce^{3+} nano-glass scintillator. *J. Eur. Ceram. Soc.* **43**, 6331-6336 (2023).
43 Liu, X. M. *et al.* Lanthanide(III)-Cu_4I_4 organic framework scintillators sensitized by cluster-based antenna for high-resolution X-ray imaging. *Adv. Mater.* **35**, 2206741 (2023).
44 Wang, Y., Chu, B., He, Q. & Xu, J. Structural and optical characterization of Ce-doped Gd_2SiO_5 films by sol–gel technique. *Appl. Surf. Sci.* **254**, 6799-6801 (2008).
45 Uchiyama, Y. *et al.* Study of energy response of Gd_2SiO_5: Ce^{3+} scintillator for the ASTRO-E hard X-ray detector. *IEEE Trans. Nucl. Sci.* **48**, 379-384 (2001).
46 Iltis, A. *et al.* Lanthanum halide scintillators: properties and applications. *Nucl. Instrum. Methods. Phys. Res. A* **563**, 359-363 (2006).
47 Higgins, W. M. *et al.* Crystal growth of large diameter $LaBr_3$:Ce and $CeBr_3$. *J. Cryst. Growth* **310**, 2085-2089 (2008).
48 Yanagida, T. *et al.* Scintillation properties of Nd^{3+}, Tm^{3+}, and Er^{3+} doped LuF_3 scintillators in the vacuum ultra violet region. *Nucl. Instrum. Methods. Phys. Res. A* **659**, 258-261 (2011).
49 Kantuptim, P. *et al.* Optical and scintillation properties of Nd-doped $Lu_2Si_2O_7$ single crystals. *J. Alloys Compd.* **860**, 158538 (2021).
50 Kantuptim, P. *et al.* Scintillation properties of Pr-doped $Lu_2Si_2O_7$ single crystal. *Radiat. Meas.* **134**, 106320 (2020).
51 Touš, J., Blažek, K., Pína, L. & Sopko, B. High-resolution imaging of biological and other objects with an X-ray digital camera. *Appl. Radiat. Isot.* **68**, 651-653 (2010).
52 Ou, Y., Zhou, W., Dorenbos, P. & Liang, H. Cationic effects on photo- and X-ray radioluminescence of $K_3RE(PO_4)_2$:Ce^{3+}/Pr^{3+} (RE = La, Gd, and Y) phosphors toward X-ray detection. *Inorg. Chem.* **62**, 6181-6188 (2023).
53 Sun, B. C. *et al.* A highly robust Ce^{3+}-doped and Gd^{3+}-mixed $KLaF_4$ nano-glass composite scintillator. *J. Mater. Chem. C* **9**, 17504-17510 (2021).
54 Sato, A. *et al.* Photoluminescence and scintillation characteristics of Bi-loaded PVK-based plastic scintillators for the high counting-rate measurement of high-energy X-rays. *RSC Adv.* **11**, 15581-15589 (2021).
55 Yanagida, T., Fujimoto, Y. & Futami, Y. Comparative study of optical and scintillation responses of $Sr_3NbGa_3Si_2O_{14}$ (SNGS) and $La_3Ta_{0.5}Ga_{5.3}Al_{0.2}O_{14}$ (LTGA) crystals. *Sens. Mater.* **27**, 247-253 (2015).
56 Esfandi, F., Saramad, S. & Shahmirzadi, M. R. Characterizing and simulation the scintillation properties of zinc oxide nanowires in AAO membrane for medical imaging applications. *J. Instrum.* **12**, P07004 (2017).
57 Chen, D. *et al.* Transmission type flat-panel X-ray source using ZnO nanowire field emitters. *Appl. Phys. Lett.* **107** (2015).
58 Xu, M. *et al.* Transient radiation imaging based on a ZnO:Ga single-crystal image

converter. *Sci. Rep.* **8**, 4178 (2018).
59 Fukushima, H., Nakauchi, D., Kato, T., Kawaguchi, N. & Yanagida, T. Luminescence and scintillation properties of Ce-doped calcium hafnate perovskite single crystals. *J. Lumin.* **250**, 119088 (2022).
60 Takahashi, K. *et al.* Photoluminescence and scintillation properties of undoped and Tl-doped Cs_2BaBr_4 crystals. *Radiat. Meas.* **132**, 106260 (2020).
61 Shimizu, M. *et al.* Luminescence and scintillation properties of Cs_3BiCl_6 crystals. *Opt. Mater.* **61**, 115-118 (2016).
62 Fujimoto, Y. *et al.* Photoluminescence, photoacoustic, and scintillation properties of Te^{4+}-doped Cs_2HfCl_6 crystals. *Mater. Res. Bull.* **105**, 291-295 (2018).
63 Fujimoto, Y. *et al.* Photoluminescence and radiation response properties of Ce^{3+}-doped $CsCaCl_3$ crystalline scintillator. *Phys. Scripta* **91**, 094002 (2016).
64 Zhao, X. *et al.* Embedding $Cs_3Cu_2I_5$ scintillators into anodic aluminum oxide matrix for high‐resolution X‐ray imaging. *Adv. Opt. Mater.* **9**, 2101194 (2021).
65 Lian, L. *et al.* Efficient and reabsorption‐free radioluminescence in $Cs_3Cu_2I_5$ nanocrystals with self‐trapped excitons. *Adv. Sci.* **7**, 2000195 (2020).
66 Gandini, M. *et al.* Efficient, fast and reabsorption-free perovskite nanocrystal-based sensitized plastic scintillators. *Nat. Nanotech.* **15**, 462-468 (2020).
67 Zhang, Y. *et al.* Metal halide perovskite nanosheet for X-ray high-resolution scintillation imaging screens. *ACS Nano* **13**, 2520-2525 (2019).
68 Ma, W. *et al.* Highly resolved and robust dynamic X‐ray imaging using perovskite glass‐ceramic scintillator with reduced light scattering. *Adv. Sci.* **8**, 2003728 (2021).
69 Zaffalon, M. L. *et al.* Extreme γ-ray radiation hardness and high scintillation yield in perovskite nanocrystals. *Nat. Photonics* **16**, 860-868 (2022).
70 Zhang, H. *et al.* Reproducible X-ray imaging with a perovskite nanocrystal scintillator embedded in a transparent amorphous network structure. *Adv. Mater.* **33**, 2102529 (2021).
71 Yang, H. *et al.* A novel scintillation screen for achieving high-energy ray detection with fast and full-color emission. *J. Mater. Chem. C* **9**, 7905-7909 (2021).
72 Yorov, K. E. *et al.* Mn^{4+}-doped fluoride nanocrystals enable high-resolution red-emitting X-ray imaging screens. *ACS Mater. Lett.* **4**, 2273-2281 (2022).
73 Fujimoto, Y. *et al.* Characterization of $CsSrCl_3$:Ce crystalline Scintillator. *Sens. Mater.* **29**, 1425-1430 (2017).
74 Su, B. B., Han, K. & Xia, Z. G. Mn^{2+}-doped Cs_2ZnBr_4 scintillator for X-ray imaging. *J. Mater. Chem. C* **11**, 8052-8061 (2023).
75 Hurley, N. *et al.* Devising novel methods for the controlled synthesis with morphology and size control of scintillator materials. *J. Mater. Chem. C* **8**, 8622-8634 (2020).
76 Fujimoto, Y., Saeki, K., Yanagida, T., Koshimizu, M. & Asai, K. Luminescence and scintillation properties of $TlCdCl_3$ crystal. *Radiat. Meas.* **106**, 151-154 (2017).
77 Wang, S. *et al.* Ruddlesden–popper perovskite nanocrystals stabilized in mesoporous silica with efficient carrier dynamics for flexible X‐ray scintillator. *Adv. Funct. Mater.* **33**, 2210765 (2023).
78 Xia, M. L. *et al.* Sub-nanosecond 2D perovskite scintillators by dielectric engineering. *Adv. Mater.* **35** (2023).

79 Kawano, N. *et al.* Scintillating organic–inorganic layered perovskite-type compounds and the gamma-ray detection capabilities. *Sci. Rep.* **7**, 14754 (2017).
80 Okazaki, K. *et al.* Scintillation properties of an organic-inorganic lead Iodide perovskite single crystal having quantum well structures. *Sens. Mater.* **34**, 575-583 (2022).
81 Zaffalon, M. L. *et al.* Zero‐dimensional Gua_3SbCl_6 crystals as intrinsically reabsorption‐free scintillators for radiation detection. *Adv. Funct. Mater.* **33**, 2305564 (2023).
82 Xu, L.-J., Lin, X., He, Q., Worku, M. & Ma, B. Highly efficient eco-friendly X-ray scintillators based on an organic manganese halide. *Nat. Commun.* **11**, 4329 (2020).
83 Xia, K. *et al.* In situ preparation of high‐quality flexible manganese‐halide scintillator films for X‐ray imaging. *Adv. Optical. Mater.* **10**, 2201028 (2022).
84 Xie, A. *et al.* Lithium-doped two-dimensional perovskite scintillator for wide-range radiation detection. *Commun. Mater.* **1**, 37 (2020).
85 Kowal, D. *et al.* PEA_2PbI_4: fast two-dimensional lead iodide perovskite scintillator with green and red emission. *Mater. Today Chem.* **29**, 101455 (2023).
86 Li, M. *et al.* Circularly polarized radioluminescence from chiral perovskite scintillators for improved X‐ray imaging. *Angew. Chem. Int. Ed.* **134**, e202208440 (2022).
87 Yuan, D., Víllora, E. G., Tominaka, S. & Shimamura, K. Distinctive Ce^{3+} luminescence from single-crystalline and glassy Ce:LaB_3O_6. *J. Mater. Chem. C* **10**, 3567-3575 (2022).
88 do Prado Labaki, H., Caixeta, F. J., Marques, N. P., Guidelli, É. J. & Gonçalves, R. R. Phase-sensitive radioluminescence and photoluminescence features in Tm^{3+} doped yttrium tantalates for cyan and white light generation. *Dalton T.* **51**, 11108-11124 (2022).
89 Nalumaga, H., Schuyt, J. J., Williams, G. V. M., Clarke, D. J. & Chong, S. V. The effect of ionising radiation on the photoluminescence and radioluminescence properties of nanoparticle and bulk $NaMgF_3$:Ce,Sm. *J. Lumin.* **228**, 117645 (2020).
90 Stellmer, S., Schreitl, M. & Schumm, T. Radioluminescence and photoluminescence of Th:CaF_2 crystals. *Sci. Rep.* **5**, 15580 (2015).
91 Schuyt, J. J. & Williams, G. V. M. Photoluminescence, radioluminescence and optically stimulated luminescence in nanoparticle and bulk $KMgF_3$(Eu). *J. Lumin.* **204**, 472-479 (2018).
92 Wahid, K., Pokhrel, M. & Mao, Y. Structural, photoluminescence and radioluminescence properties of Eu^{3+} doped $La_2Hf_2O_7$ nanoparticles. *J. Solid State Chem.* **245**, 89-97 (2017).
93 Ayvacıklı, M. *et al.* Radioluminescence and photoluminescence characterization of Eu and Tb doped barium stannate phosphor ceramics. *J. Alloys Compd.* **590**, 417-423 (2014).
94 Williams, G. V. M., Janssens, S., Gaedtke, C., Raymond, S. G. & Clarke, D. Observation of photoluminescence and radioluminescence in Eu and Mn doped $NaMgF_3$ nanoparticles. *J. Lumin.* **143**, 219-225 (2013).
